# Supplementary material for: Response of benthic macroinvertebrates to dam removal in the restoration of the Boardman River, Michigan, USA
Source: PLoS One. 2021 May 19;16(5):e0245030. doi: 10.1371/journal.pone.0245030 (PMC8133408; doi:10.1371/journal.pone.0245030)
Supplement: S2 File — (DOCX) [file pone.0245030.s002.docx]

**S2. Table of the three most abundant families at each site in each year.** Total abundances of benthic macroinvertebrates summed from six Surber samples (0.093 m2 per sample) at sites along the Boardman River, Grand Traverse and Kalkaska counties, Michigan (USA). In cases where there were equal numbers for any of most abundant families, the next most abundant families were included. Italicized families indicate tolerant taxa (families Simuliidae and Chironomidae, and subclass Oligochaeta), and bold families indicate sensitive taxa (Ephemeroptera, Plecoptera, and Trichoptera). Superscripts were added: A = phylum Annelida, C = order Coleoptera, D = order Diptera, E = order Ephemeroptera, G = class Gastropoda, I = order Isopoda, N = phylum Nematomorpha, R = class Arachnida, T = order Trichoptera.

| **Year** | **Site** | **Most abundant taxa** |
| --- | --- | --- |
| 2011 | GHU  GHL  SHU | **Ephemerellidae**^E^ (122), *Chironomidae*^D^ (65), Elmidae^C^ (58)  **Ephemerellidae**^E^ (16), *Chironomidae*^D^ (10), **Brachycentridae**^T^ (10), Elmidae^C^ (6)  Elmidae^C^ (115), *Simuliidae*^D^ (35), **Helicopsychidae**^T^ (26) |
|  | LP | *Chironomidae*^D^ (48), **Baetidae**^E^ (14), **Brachycentridae**^T^ (11), **Ephemerellidae**^E^ (11) |
| 2012 | GHU  GHL  BBR  14P  WYR  SUM  SHU | **Ephemerellidae**^E^ (128), Elmidae^C^ (41), *Chironomidae*^D^ (21)  **Ephemerellidae**^E^ (7), Hydrachnidae^R^ (6), **Brachycentridae**^T^ (3)  Elmidae^C^ (80), **Ephemerellidae**^E^ (68), **Hydropsychidae**^T^ (33)  **Ephemerellidae**^E^ (90), Elmidae^C^ (63), **Hydropsychidae**^T^ (56)  Elmidae^C^ (32), **Ephemerellidae**^E^ (11), **Brachycentridae**^T^ (9)  Elmidae^C^ (76), Athericidae^D^ (48), **Brachycentridae**^T^ (15)  Elmidae^C^ (105), *Simuliidae*^D^ (47), **Ephemerellidae**^E^ (46) |
|  | LP | **Helicopsychidae**^T^ (6), *Chironomidae*^D^ (5), **Brachycentridae**^T^ (3) |
| 2013 | GHU  GHL  BBU  BBM  BBL  BBR  14P  WYR  SUM  SHU | **Ephemerellidae**^E^ (107), Elmidae^C^ (27), Hydrobiidae^G^ (25)  Hydrobiidae^G^ (184), *Chironomidae*^D^ (10), *Oligochaeta*^A^ (4)  Hydrobiidae^G^ (29), **Ephemerellidae**^E^ (27), *Chironomidae*^D^ (19)  *Chironomidae*^D^ (262), **Ephemerellidae**^E^ (60), Hydrobiidae^G^ (44)  *Chironomidae*^D^ (76), **Baetidae**^E^ (33), **Ephemerellidae**^E^ (16)  *Chironomidae*^D^ (27), *Simuliidae*^D^ (13), Hydrobiidae^G^ (6)  Elmidae^C^ (41), *Chironomidae*^D^ (40), *Oligochaeta*^A^ (14)  *Chironomidae*^D^ (40), Elmidae^C^ (24), Athericidae^D^ (9)  *Simuliidae*^D^ (93), *Chironomidae*^D^ (41), Elmidae^C^ (20)  Elmidae^C^ (48), *Chironomidae*^D^ (34), **Brachycentridae**^T^ (19) |
|  | LP | *Chironomidae*^D^ (14), *Simuliidae*^D^ (11), **Brachycentridae**^T^ (3), **Baetidae**^E^ (3), Elmidae^C^ (3) |
| 2014 | GHU  GHL  BBU  BBM  BBL  BBR  14P  WYR  SUM  SHU | **Ephemerellidae**^E^ (74), Elmidae^E^ (46), Gordidae^N^ (22)  Hydrobiidae^G^ (61), **Heptageniidae**^E^ (32), **Baetidae**^E^ (14)  *Chironomidae*^D^ (123), **Baetidae**^E^ (48), Hydrobiidae^G^ (34)  Hydrobiidae^G^ (66), *Chironomidae*^D^ (58), Gordidae^N^ (51)  **Heptageniidae**^E^ (41), *Chironomidae*^D^ (41), **Baetidae**^E^ (33)  **Heptageniidae**^E^ (35), Athericidae^D^ (25), **Ephemerellidae**^E^ (17)  **Ephemerellidae**^E^ (61), Elmidae^C^ (60), **Baetidae**^E^ (52)  Elmidae^C^ (53), **Ephemerellidae**^E^ (41), **Baetidae**^E^ (28)  **Baetidae**^E^ (137), Elmidae^C^ (113), **Ephemerellidae**^E^ (51)  **Baetidae**^E^ (21), Elmidae^C^ (17), Athericidae^D^ (14) |
|  | LP | **Baetidae**^E^ (49), *Chironomidae*^D^ (33), **Ephemerellidae**^E^ (7) |
| 2015 | GHU  GHL  BBU  BBM  BBL  BBR  14P  WYR  SUM  SHU | Hydrobiidae^G^ (68), **Ephemerellidae**^E^ (51), *Chironomidae*^D^ (34)  **Heptageniidae**^E^ (35), **Ephemerellidae**^E^ (33), Hydrobiidae^G^ (19)  **Ephemerellidae**^E^ (80), *Chironomidae*^D^ (77), Hydrobiidae^G^ (26)  **Ephemerellidae**^E^ (134), *Chironomidae*^D^ (61), Hydrobiidae^G^ (25)  **Ephemerellidae**^E^ (126), Hydrobiidae^G^ (56), Elmidae^C^ (22)  **Ephemerellidae**^E^ (81), Hydrobiidae^G^ (60), Elmidae^C^ (17)  Hydrobiidae^G^ (108), **Ephemerellidae**^E^ (74), *Chironomidae*^D^ (46)  **Ephemerellidae**^E^ (48), *Oligochaeta*^A^ (27), *Chironomidae*^D^ (26)  *Chironomidae*^D^ (28), **Ephemerellidae**^E^ (24), Hydrobiidae^G^ (21)  *Chironomidae*^D^ (25), Hydrobiidae^G^ (21), **Ephemerellidae**^E^ (20) |
| 2016 | GHU  GHL  BBR  14P  WYR  SUM  SHU | Hydrobiidae^G^ (422), **Ephemerellidae**^E^ (44), Elmidae^C^ (34)  Hydrobiidae^G^ (779), **Baetidae**^E^ (10), **Ephemerellidae**^E^ (9), Elmidae^C^ (9)  Hydrobiidae^G^ (465), **Ephemerellidae**^E^ (52), Elmidae^C^ (35)  Hydrobiidae^G^ (1829), Elmidae^C^ (124), **Ephemerellidae**^E^ (38)  Hydrobiidae^G^ (438), Elmidae^C^ (26), Hydrachnidae^R^ (21)  Hydrobiidae^G^ (302), Elmidae^C^ (97), **Ephemerellidae**^E^ (49)  Hydrobiidae^G^ (400), **Ephemerellidae**^E^ (49), **Brachycentridae**^T^ (18) |
