# Supplementary material for: Response of benthic macroinvertebrates to dam removal in the restoration of the Boardman River, Michigan, USA
Source: PLoS One. 2021 May 19;16(5):e0245030. doi: 10.1371/journal.pone.0245030 (PMC8133408; doi:10.1371/journal.pone.0245030)

Boardman River Dam Removal Analysis

# function to calculate gini-simpson diversity
gsdi<-function(x,replace=FALSE) {
 if(is.numeric(x)!=TRUE) stop("x must be numeric")
 if(is.logical(replace)!=TRUE) stop("replace must be logical")
 if(replace==TRUE) {
 return ( 1-sum((x/sum(x,na.rm=TRUE))^2,na.rm=TRUE) )
 } else
 {return(1-sum(x*(x-1),na.rm=TRUE)/(sum(x,na.rm=TRUE)*(sum(x,na.rm=TRUE)-1)))} } # calculate GSI

### Below is the code used to load and clean the data ----

### First import ----
library(readxl)
fam_data <- read_excel("~/Dropbox/Boardman River/Boardman River Data 30 March 2018.xlsx",
 sheet = "Family Level Data, 3-30-2018", col_names = FALSE, range = "A9:CH66")
# skip info tables below the main data
names(fam_data)<-c("Year","Site",names(read_excel("~/Dropbox/Boardman River/Boardman River Data 30 March 2018.xlsx", range="C5:BO5")),names(read_excel("~/Dropbox/Boardman River/Boardman River Data 30 March 2018.xlsx", range="BP8:Cg8")))
fam_data<-fam_data[-c(3,68)] # remove some NA columns
names(fam_data)[c(3,52:53,57,64)]<-c("Unk_Colembola","Unk_Oligochaeta","Unk_Hirudinae","unk_Bivalvia","Unk_Gastropoda")
fam_abundance<-fam_data[,1:66] # just the abundance data

# taxonomic and grouping info for families
# FFGs from Merrit and Cummins 1996
# SH = Shredders; CG = Collectors/Gatherers; CF = Collectors/Filterers; SC = Scrapers;
# PR = Predators; PI = Piercers
# Tolerance Vals from Bouchard et al 2004; 0:3|0:4 = sensitive, 6:10|7:10 = tolerant
fam_info<-data.frame(t(read_excel("~/Dropbox/Boardman River/Boardman River Data 30 March 2018.xlsx", range="d2:BO8",col_names = FALSE)))
names(fam_info)<-c("Phylum","Class","Order","Family","Beh_Grp","Func_Feeding_Grp","Tol_Lev")
row.names(fam_info)<-names(fam_abundance)[-(1:2)]

insects<-which(fam_info$Class == "Insecta")+2 # columns for insects
ept<-with(fam_info,which(Order=="Ephemeroptera"|Order=="Plecoptera"|Order=="Trichoptera"))+2

fam_data<-fam_data[,c(1,2,67:70)] # keep site/year info and; dump suspect summaries from the Excel file
names(fam_data)[3]<-"Dist" # Distance below dam (m) -neg values are above
# Totals
fam_data$Total_inverts <- rowSums(fam_abundance[,-c(1:2)]) #totals
fam_data$Total_inverts_noHydro <- rowSums(fam_abundance[,-c(1:2,59)]) #totals w/o Hydrobiidae
fam_data$Total_insects <- rowSums(fam_abundance[insects]) # total insects
fam_data$Total_EPT <- rowSums(fam_abundance[ept]) # total ept
# % EPT
fam_data$percent_EPT <- with(fam_data, Total_EPT/Total_inverts)
fam_data$percent_EPT_noHydro <- with(fam_data, Total_EPT/Total_inverts_noHydro)
fam_data$percent_EPT_insects <- with(fam_data, Total_EPT/Total_insects)
# Density (based on sampled area of 0.55800 m^2)
fam_data$Density_inverts<-fam_data$Total_inverts/0.55742
fam_data$Density_inverts_noHydro<-fam_data$Total_inverts_noHydro/0.55742
fam_data$Density_insects <- fam_data$Total_insects/0.55742
fam_data$Density_EPT <- fam_data$Total_EPT/0.55742
# Richness
fam_data$Richness<-apply(X = fam_abundance[,-c(1:2)],MARGIN = 1,FUN = function(x) sum(x>0))
fam_data$Richness_noHydro<-apply(X = fam_abundance[,-c(1:2,59)],MARGIN = 1,FUN = function(x) sum(x>0))
# diversity index
fam_data$GSI <- apply(X = fam_abundance[,-c(1:2)],MARGIN = 1,FUN = gsdi)
fam_data$GSI_noHydro <- apply(X = fam_abundance[,-c(1:2,59)],MARGIN = 1,FUN = gsdi)
fam_data$GSI_insects <- apply(X = fam_abundance[,insects],MARGIN = 1,FUN = gsdi)
fam_data<-fam_data[-which(fam_data$Site=="BBR" & fam_data$Year=="2011"),]
# remove BBR in 2011, a mussel bed was sampled

## export
# write.table(fam_data,"FamilyDataSummary.csv",sep=",",row.names = FALSE)

## set up multivariate data ----
mv_fam<-fam_abundance
# fam_abundance is Site + year + abundances of 64 taxa
mv_fam<-mv_fam[-which(mv_fam$Site=="BBR" & mv_fam$Year=="2011"),]
# remove BBR in 2011, a mussel bed was sampled
mv_fam$Site.Yr<-with(mv_fam,paste(Site,substr(Year,start=3,stop=4),sep="-"))
# add site*yr variable
# all(mv_fam$Site==fam_data$Site) # TRUE
# all(mv_fam$Year==fam_data$Year) # TRUE
mv_fam$Dist<-fam_data$Dist # can only do b/c the above 2 tests are true
# the following creates impact class variable:
mv_fam$Impact<-0 #upstream
mv_fam$Impact[which(mv_fam$Dist>0 & mv_fam$Year>2012)]<- 2 #22 downstream, after
mv_fam$Impact[which(mv_fam$Dist<0 & mv_fam$Dist>-5000)]<-3 #9; new sites
mv_fam$Impact[which(mv_fam$Dist>0 & mv_fam$Year<=2012)]<- 1 # 15 dwonstream, before
mv_fam$Impact<-factor(mv_fam$Impact,labels=c("Upstream","Downstream-before","Downstream-after","New"))
# Now col 1:2 and 67:69 are non-numeric data
mv_fam2<-as.data.frame(mv_fam)
row.names(mv_fam2)<-mv_fam2$Site.Yr
mv_fam2<-as.matrix(mv_fam2[,-c(1,2,67:69)]) # only numeric
# mv_fam2 is a matrix of all taxa, rare taxa not removed. Col 57 is hydrobiidae
rare_fam_abundance<-mv_fam[,c(1:2,which(colSums(mv_fam[,3:66])<6)+2)] # should be 30 columns
mv_fam<-mv_fam[,-(which(colSums(mv_fam[,3:66])<6)+2)] # 36 families
cat("The following family/taxa will be removed")
names(rare_fam_abundance)[-(1:2)] # print taxa removed
mv_fam.taxa<-as.matrix(mv_fam[,-c(1:2,39:41)])
# mv_fam.taxa is a matrix of prevalence for families for each site
rownames(mv_fam.taxa)<- mv_fam$Site.Yr # 57x36

# repeat the last steps for all sites except "new" sites
mv_fam.taxa2<-as.matrix(mv_fam[-which(mv_fam$Impact=="New"),-c(1:2,39:41)])
# a matrix of prevalence for orders for each site except "new"
# which(colSums(mv_fam.taxa2)<=5) # 2 taxa w/ 5 or fewer
# which(names(fam_abundance)%in%c("Limnephelidae","Gammaridae"))
rare_fam_abundance<-cbind(rare_fam_abundance,fam_abundance[-which(fam_abundance$Site=="BBR" & fam_abundance$Year=="2011"),which(names(fam_abundance)%in%c("Limnephelidae","Gammaridae"))])
# add these 2 records to "rare families"
# dim(mv_fam.taxa2) # 48x36
mv_fam.taxa2<-mv_fam.taxa2[,-which(colSums(mv_fam.taxa2)<=5)]
# dim(mv_fam.taxa2) # 48x34
rownames(mv_fam.taxa2)<- mv_fam$Site.Yr[-which(mv_fam$Impact=="New")]

#### Save Data Sets:
# save(list = c("fam_data", "fam_abundance","mv_fam","mv_fam.taxa","mv_fam.taxa2","mv_fam2"), file = "BoardmanDataExport.Rdata")

## Boardman River Community Data

### fam_data ----
# The original data imported as fam_data, but then the abundance data is split off, and fam_data is used to store the recalculated summary variables (See code below)

### fam_abundance ----
# Only the abundance data from fam_data, with site & year in the the first 2 columns
# 58x66
# 64 families, of which 28 have 5 or fewer records (after BBR 2011(row 9) is removed from the data), as shown by:
# colSums(fam_abundance[-9,-(1:2)])[which(colSums(fam_abundance[,-(1:2)])<6)]

### mv_fam ----
# data frame, 57x41, multivariate community data at the family level, with the 36 taxa (with at least 6 count)
# rare taxa removed, BBR 2011 removed, and impact classes, and site year included.

### mv_fam.taxa ----
# matrix, 57x36 of prevalence for families for each site, rare families are removed
# "new" sites are included
# rows 1:6 are 2008-2010 data for SHU and LP; we've been excluding those.
# column 30 is Hydrobiidae, col 35 is Unk_Gastropoda, which may or may not be Hydrobiidae. I have not been excluding Unk_Gastropoda

### mv_fam.taxa2 ----
# matrix, 48*34 of prevalencce for families at the sites, excluding the "new" (formerly impounded) sites.
# rows 1:6 are 2008-2010 data for SHU and LP; we've been excluding those.
# column 28 is Hydrobiidae, col 33 is Unk_Gastropoda, which may or may not be Hydrobiidae. I have not been excluding Unk_Gastropoda

### rare_fam_abundance ----
# data.frame, 57x32 all the records for the families removed from analysis for low occurence rate. These were found at 24/57 site*years

#### ----

#### Load the Data:
load("BoardmanDataExport.Rdata") # file must be in your working directory.

Extent of the data. The sites “NBL”, “NBM”, and “NBU” were only sampled in 2014, and we will exclude them from the data. The 2011 sample at “BBR” inadvertently sampled a mussel bed, so this will also be excluded.

# in order to rerun the code, we'll rename the variables in fam_data to match those in dam
fam_data2<-fam_data # copy the data
names(fam_data2)[c(4:11)]<-c("Lat","Long","Before.After","Total.MI","MI.noHydrobiidae","Total.Insects","Total.EPT","EPT.pct")
names(fam_data2)[12:19]<-c("EPT.pct.noHyd","EPT.pct.insect","Inverts.den","Inverts.den2","Insect.den","EPT.den","Richness.taxa","Richness.taxa2")
names(fam_data2)[c(21)]<-c("GSI.all2") # GSI w/o hydrobiid snails

# original_dam_data<-dam # keep a copy of the original data
dam<-fam_data2 # push fam_data with dam names into dam
rm(fam_data2) # don't need the copy


fam_data$Impact<-0
fam_data$Impact[which(fam_data$Dist>0 & fam_data$Year>2012)]<- 2 #22 downstream, after
fam_data$Impact[which(fam_data$Dist<0 & fam_data$Dist>-5000)]<-3 #9; new sites
fam_data$Impact[which(fam_data$Dist>0 & fam_data$Year<=2012)]<- 1 # 15 downstream, before
fam_data$Impact<-factor(fam_data$Impact,labels=c("Upstream","Downstream-before","Downstream-after", "New"))

dam$Impact<-fam_data$Impact
dam$Impact2<-dam$Impact
levels(dam$Impact2)<-c("N","N","I","New")

# with(dam,table(Site,Year)) #
 # distance from dam
site.distances<-aggregate(dam$Dist,by=dam[2],FUN=max)
names(site.distances)[2]<-"Distance"
 # site.distances
# years by site
site.years<-aggregate(dam$Year,by=dam[2],min)
site.years[,3]<-aggregate(dam$Year,by=dam[2],max)[,2]
site.years[,4]<-aggregate(dam$Year,by=dam[2],length)[,2]
site.years[,5]<-site.distances[,2]
names(site.years)[2:5]<-c("First","Last","n","Distance")
site.years<-site.years[order(site.years$Distance),]
site.years$Type<-c(rep("Upstream",2),rep("New",3),rep("Downstream",6))
library(knitr)
kable(site.years,row.name=FALSE)

| Site | First | Last | n | Distance | Type |
| --- | --- | --- | --- | --- | --- |
| GHU | 2011 | 2016 | 6 | -6100 | Upstream |
| GHL | 2011 | 2016 | 6 | -5260 | Upstream |
| BBU | 2013 | 2015 | 3 | -4180 | New |
| BBM | 2013 | 2015 | 3 | -3470 | New |
| BBL | 2013 | 2015 | 3 | -182 | New |
| BBR | 2012 | 2016 | 5 | 1060 | Downstream |
| 14P | 2012 | 2016 | 5 | 2350 | Downstream |
| WYR | 2012 | 2016 | 5 | 6280 | Downstream |
| SUM | 2012 | 2016 | 5 | 8610 | Downstream |
| SHU | 2008 | 2016 | 9 | 11240 | Downstream |
| LP | 2008 | 2014 | 7 | 20460 | Downstream |

# 37 "Downstream", 21 "Upstream", 22 "Impacted", 38 "Non-impacted"

###### Table 1. Sites and the years for which data was collected. Negative distances are “upstream” of the former dam. Distances further upstream than ~ -5200 were above the (former) impoundment.

Not all sites are sampled in all years.

### Refit Linear Models

- Fit models for only Invert Density, Taxon Richness, and EPT%
- 2011-2016, Upsteam and Down-before and Down-after (exclude “New” sites)
- 2013-2016, Upstream, Downstream-after, and New

Here we are looking for differences site type*year (Impacted or Non, New sites excluded) so we have 10 groups - Impacted sites in 2013:2016 and Non-impacted sites in 2011:2016. We fit a model with our response as a function of site type*year and with actual site as a random effect (so that differences between sites are included in the model). This is a work around - we can’t fit Site Type and Year as factors in the model and include an interaction, since there are no impacted sites in 2011 or 2012.

require(nlme)
require(multcomp)

dam$Site.Yr<-factor(paste(dam$Site,dam$Year,sep=""))
dam$Impact.Yr<-factor(paste(dam$Impact2,dam$Year,sep=""))
damsub<-droplevels(subset(dam,Impact2!="New"&Year>=2011))
damsub$Year<-factor(damsub$Year)
dam$Year<-factor(dam$Year)

#### Impacted vs Non-impacted

At general level we can compare “Impacted” with “Non-impacted” sites. We’ll exclude “new” sites here since they might have a different type of impact. Here we define “Impacted” as “exposed to sediment and scouring after the removal of the dam”

m1<-lme(Inverts.den2~Impact2,random=~1|Site/Year,data=droplevels(subset(dam,Impact2!="New")))
anova(m1)

## numDF denDF F-value p-value
## (Intercept) 1 39 47.37957 <.0001
## Impact2 1 39 1.66487 0.2045

# summary(glht(m1,linfct=mcp(Impact2="Tukey")))
# plot(m1) # unexplained variation in residuals but not heteroschedatic

No differences in Invertebrate density associated with Impact/No-impact.

m1<-lme(Richness.taxa2~Impact2,random=~1|Site/Year,data=droplevels(subset(dam,Impact2!="New")))
anova(m1)

## numDF denDF F-value p-value
## (Intercept) 1 39 337.9235 <.0001
## Impact2 1 39 0.1006 0.7528

# summary(glht(m1,linfct=mcp(Impact2="Tukey")))
# plot(m1) # unexplained variation in residuals but not heteroschedatic

Taxon richness did not differ between impacted and non-impacted sites.

m1<-lme(EPT.pct.noHyd~Impact2,random=~1|Site/Year,data=droplevels(subset(dam,Impact2!="New")))
anova(m1)

## numDF denDF F-value p-value
## (Intercept) 1 39 311.75030 <.0001
## Impact2 1 39 0.70533 0.4061

# summary(glht(m1,linfct=mcp(Impact2="Tukey")))
# plot(m1) # unexplained variation in residuals but not heteroschedatic

EPT % did not differ between impacted and non-impacted sites.

These three summary variables did not differ between these two categories of site*year

ffg$Imp<-ffg$Impact
levels(ffg$Imp)<-c("N","I","N","New")
m1<-lme(CG~Imp,random=~1|Site/Year,data=droplevels(subset(ffg,Imp!="New")))
anova(m1)

## numDF denDF F-value p-value
## (Intercept) 1 39 58.09154 <.0001
## Imp 1 39 0.10849 0.7436

# summary(glht(m1,linfct=mcp(Impact2="Tukey")))
# plot(m1) # unexplained variation in residuals but not heteroschedatic

m1<-lme(CF~Imp,random=~1|Site/Year,data=droplevels(subset(ffg,Imp!="New")))
anova(m1)

## numDF denDF F-value p-value
## (Intercept) 1 39 17.457419 0.0002
## Imp 1 39 1.552695 0.2202

# summary(glht(m1,linfct=mcp(Impact2="Tukey")))
# plot(m1) # unexplained variation in residuals but not heteroschedatic

m1<-lme(SC.noHydro~Imp, random=~1|Site/Year,data=droplevels(subset(ffg,Imp!="New")))
anova(m1)

## numDF denDF F-value p-value
## (Intercept) 1 39 13.975536 0.0006
## Imp 1 39 6.494119 0.0149

# summary(glht(m1,linfct=mcp(Impact2="Tukey")))
# plot(m1) # unexplained variation in residuals but not heteroschedatic

m1<-lme(PR~Imp, random=~1|Site/Year,data=droplevels(subset(ffg,Imp!="New")))
anova(m1)

## numDF denDF F-value p-value
## (Intercept) 1 39 25.260969 <.0001
## Imp 1 39 0.167553 0.6845

# summary(glht(m1,linfct=mcp(Impact2="Tukey")))
# plot(m1) # unexplained variation in residuals but not heteroschedatic

No difference between Impacted and Non-impacted sites in abundance of collector/gatherers, collector/filterers, or Predators.

m1<-lme(SH~Imp,random=~1|Site/Year,data=droplevels(subset(ffg,Imp!="New")))
anova(m1)

## numDF denDF F-value p-value
## (Intercept) 1 39 33.33561 <.0001
## Imp 1 39 5.89470 0.0199

summary(m1)

## Linear mixed-effects model fit by REML
## Data: droplevels(subset(ffg, Imp != "New"))
## AIC BIC logLik
## 325.9121 335.0553 -157.956
##
## Random effects:
## Formula: ~1 | Site
## (Intercept)
## StdDev: 0.0002068803
##
## Formula: ~1 | Year %in% Site
## (Intercept) Residual
## StdDev: 6.999647 0.03994761
##
## Fixed effects: SH ~ Imp
## Value Std.Error DF t-value p-value
## (Intercept) 3.576923 1.372766 39 2.605632 0.0129
## ImpI 4.923077 2.027710 39 2.427900 0.0199
## Correlation:
## (Intr)
## ImpI -0.677
##
## Standardized Within-Group Residuals:
## Min Q1 Med Q3 Max
## -0.006930160 -0.002916312 -0.001254328 0.001364081 0.019975168
##
## Number of Observations: 48
## Number of Groups:
## Site Year %in% Site
## 8 48

# plot(m1) # unexplained variation in residuals but not heteroschedatic

m1<-lme(SC.noHydro~Imp, random=~1|Site/Year,data=droplevels(subset(ffg,Imp!="New")))
anova(m1)

## numDF denDF F-value p-value
## (Intercept) 1 39 13.975536 0.0006
## Imp 1 39 6.494119 0.0149

summary(m1)

## Linear mixed-effects model fit by REML
## Data: droplevels(subset(ffg, Imp != "New"))
## AIC BIC logLik
## 494.9788 504.122 -242.4894
##
## Random effects:
## Formula: ~1 | Site
## (Intercept)
## StdDev: 31.8598
##
## Formula: ~1 | Year %in% Site
## (Intercept) Residual
## StdDev: 36.10529 15.11463
##
## Fixed effects: SC.noHydro ~ Imp
## Value Std.Error DF t-value p-value
## (Intercept) 64.41355 14.32253 39 4.497357 0.0001
## ImpI -35.42149 13.89974 39 -2.548356 0.0149
## Correlation:
## (Intr)
## ImpI -0.47
##
## Standardized Within-Group Residuals:
## Min Q1 Med Q3 Max
## -0.74601116 -0.22902925 -0.04224485 0.13225303 1.46389744
##
## Number of Observations: 48
## Number of Groups:
## Site Year %in% Site
## 8 48

# plot(m1) # unexplained variation in residuals but not heteroschedatic

Impacted plots have greater abundance of Shredders (p=0.02), and a lower abundance of scrapers (p=0.015) than non-impacted sites.

#### Impact classes

Since the general groupings might obscure differences between downstream sites before and after dam removal, we’ll use a finer classification. We can categorize sites into 4 groups according to how the dam impacts them: Upstream, Downstream (before dam removal), Downstream (afer dam removal), and New. Here we’re trying to predict community parameters as a function of the impact group with site and year as random effects.

dam$Impact3<-dam$Impact
levels(dam$Impact3)<-c("U","D-b","D-a","N") # shorthand for Impact
m1<-lme(Inverts.den2~Impact3,random=~1|Site/Year,data=dam)
anova(m1)

## numDF denDF F-value p-value
## (Intercept) 1 43 76.65464 <.0001
## Impact3 3 43 1.66943 0.1877

# summary(glht(m1,linfct=mcp(Impact3="Tukey")))
# plot(m1) # unexplained variation in residuals but not heteroschedatic

No significant differences in Invertebrate density across these groups.

m1<-lme(Richness.taxa2~Impact3,random=~1|Site/Year,data=dam)
anova(m1)

## numDF denDF F-value p-value
## (Intercept) 1 43 486.8525 <.0001
## Impact3 3 43 0.6592 0.5817

# summary(glht(m1,linfct=mcp(Impact3="Tukey")))
# plot(m1) # unexplained variation in residuals but not heteroschedatic

No significant differences in Taxon richness across these groups.

m1<-lme(EPT.pct.noHyd~Impact3,random=~1|Site/Year,data=dam)
anova(m1)

## numDF denDF F-value p-value
## (Intercept) 1 43 407.2521 <.0001
## Impact3 3 43 1.3311 0.2767

# summary(glht(m1,linfct=mcp(Impact3="Tukey")))
# plot(m1) # unexplained variation in residuals but not heteroschedatic

No significant differences in EPT % across these groups.

These three summary variables did not show any differences associated with the four categories of site*year.

Turning to the functional feeding groups:

m1<-lme(CG~Impact,random=~1|Site/Year,data=ffg)
anova(m1)

## numDF denDF F-value p-value
## (Intercept) 1 43 76.83888 <.0001
## Impact 3 43 3.54834 0.0221

summary(glht(m1,linfct=mcp(Impact="Tukey")))

##
## Simultaneous Tests for General Linear Hypotheses
##
## Multiple Comparisons of Means: Tukey Contrasts
##
##
## Fit: lme.formula(fixed = CG ~ Impact, data = ffg, random = ~1 | Site/Year)
##
## Linear Hypotheses:
## Estimate Std. Error z value Pr(>|z|)
## Downstream-after - Upstream == 0 -2.704 29.745 -0.091 0.99972
## Downstream-before - Upstream == 0 6.943 32.122 0.216 0.99636
## New - Upstream == 0 92.444 35.413 2.610 0.04362
## Downstream-before - Downstream-after == 0 9.647 23.893 0.404 0.97727
## New - Downstream-after == 0 95.148 30.561 3.113 0.00959
## New - Downstream-before == 0 85.501 32.878 2.601 0.04443
##
## Downstream-after - Upstream == 0
## Downstream-before - Upstream == 0
## New - Upstream == 0 *
## Downstream-before - Downstream-after == 0
## New - Downstream-after == 0 **
## New - Downstream-before == 0 *
## ---
## Signif. codes: 0 '***' 0.001 '**' 0.01 '*' 0.05 '.' 0.1 ' ' 1
## (Adjusted p values reported -- single-step method)

# plot(m1) # unexplained variation in residuals but not heteroschedatic

Abundance of Collector/Gatherers was higher in the new sites compared to all the other sites (p<0.045), but the difference was strong for New sites vs Downstream-before sites (p<0.01).

Same general pattern, but weaker significance, if we divide upstream sites into ‘upstream-before’ and ‘upstream-after’

m1<-lme(CF~Impact,random=~1|Site/Year,data=ffg)
anova(m1)

## numDF denDF F-value p-value
## (Intercept) 1 43 34.07555 <.0001
## Impact 3 43 1.96998 0.1327

# summary(glht(m1,linfct=mcp(Impact2="Tukey")))
# plot(m1) # unexplained variation in residuals but not heteroschedatic

m1<-lme(SH~Impact,random=~1|Site/Year,data=ffg)
anova(m1)

## numDF denDF F-value p-value
## (Intercept) 1 43 50.27586 <.0001
## Impact 3 43 2.59035 0.0651

# summary(glht(m1,linfct=mcp(Impact2="Tukey")))
# plot(m1) # unexplained variation in residuals but not heteroschedatic

m1<-lme(PR~Impact,random=~1|Site/Year,data=ffg)
anova(m1)

## numDF denDF F-value p-value
## (Intercept) 1 43 32.88318 <.0001
## Impact 3 43 0.09378 0.9631

# summary(glht(m1,linfct=mcp(Impact2="Tukey")))
# plot(m1) # unexplained variation in residuals but not heteroschedatic

Abundance of Collector/Filterers, and predators did not differ between impacted and non-impacted sites. There was a marginal difference for Shredders (p=0.065).

m1<-lme(SC.noHydro~Impact,random=~1|Site/Year,data=ffg)
anova(m1)

## numDF denDF F-value p-value
## (Intercept) 1 43 22.461997 <.0001
## Impact 3 43 3.844238 0.0159

summary(glht(m1,linfct=mcp(Impact="Tukey")))

##
## Simultaneous Tests for General Linear Hypotheses
##
## Multiple Comparisons of Means: Tukey Contrasts
##
##
## Fit: lme.formula(fixed = SC.noHydro ~ Impact, data = ffg, random = ~1 |
## Site/Year)
##
## Linear Hypotheses:
## Estimate Std. Error z value Pr(>|z|)
## Downstream-after - Upstream == 0 7.497 23.664 0.317 0.9884
## Downstream-before - Upstream == 0 48.981 24.759 1.978 0.1870
## New - Upstream == 0 -7.222 27.271 -0.265 0.9932
## Downstream-before - Downstream-after == 0 41.484 13.588 3.053 0.0109
## New - Downstream-after == 0 -14.719 22.386 -0.658 0.9089
## New - Downstream-before == 0 -56.203 23.541 -2.387 0.0742
##
## Downstream-after - Upstream == 0
## Downstream-before - Upstream == 0
## New - Upstream == 0
## Downstream-before - Downstream-after == 0 *
## New - Downstream-after == 0
## New - Downstream-before == 0 .
## ---
## Signif. codes: 0 '***' 0.001 '**' 0.01 '*' 0.05 '.' 0.1 ' ' 1
## (Adjusted p values reported -- single-step method)

# plot(m1) # not bad.

Abundance of scrapers in downstream sites before dam removal was greater than after dam removal (p=0.011) - this is with Hydrobiids excluded.

#### Impacted vs Non-impacted by years

If we want to drill down to actual year level data, to see if there is a short-term effect of dam removal, we should compared Impacted (downstream, after removal) sites with non-impacted (upstream and downstream, before) removal sites for each year. Since this already creates 10 groups, we’ll drop the new sites from this analysis.

m1<-lme(Inverts.den2~Impact.Yr,random=~1|Site,data=damsub)
anova(m1) #

## numDF denDF F-value p-value
## (Intercept) 1 25 43.23674 <.0001
## Impact.Yr 9 25 0.73676 0.6723

# summary(glht(m1,linfct=mcp(Impact.Yr="Tukey")))
# plot(m1) # residuals show some heteroschedasticity, largely due to 1 outlier.

No differences in invertebrate density when Hydrobiids were excluded from the analysis.

m1<-lme(Inverts.den~Impact.Yr,random=~1|Site,data=damsub)
anova(m1) #

## numDF denDF F-value p-value
## (Intercept) 1 25 36.75585 <.0001
## Impact.Yr 9 25 4.64834 0.0011

summary(glht(m1,linfct=mcp(Impact.Yr="Tukey")))

## Warning in RET$pfunction("adjusted", ...): Completion with error > abseps

## Warning in RET$pfunction("adjusted", ...): Completion with error > abseps

## Warning in RET$pfunction("adjusted", ...): Completion with error > abseps

## Warning in RET$pfunction("adjusted", ...): Completion with error > abseps

## Warning in RET$pfunction("adjusted", ...): Completion with error > abseps

## Warning in RET$pfunction("adjusted", ...): Completion with error > abseps

## Warning in RET$pfunction("adjusted", ...): Completion with error > abseps

## Warning in RET$pfunction("adjusted", ...): Completion with error > abseps

## Warning in RET$pfunction("adjusted", ...): Completion with error > abseps

##
## Simultaneous Tests for General Linear Hypotheses
##
## Multiple Comparisons of Means: Tukey Contrasts
##
##
## Fit: lme.formula(fixed = Inverts.den ~ Impact.Yr, data = damsub, random = ~1 |
## Site)
##
## Linear Hypotheses:
## Estimate Std. Error z value Pr(>|z|)
## I2014 - I2013 == 0 149.199 259.604 0.575 0.9999
## I2015 - I2013 == 0 141.667 273.189 0.519 1.0000
## I2016 - I2013 == 0 1385.972 273.189 5.073 <0.01 ***
## N2011 - I2013 == 0 126.534 297.496 0.425 1.0000
## N2012 - I2013 == 0 132.007 244.347 0.540 0.9999
## N2013 - I2013 == 0 196.143 382.825 0.512 1.0000
## N2014 - I2013 == 0 110.032 382.825 0.287 1.0000
## N2015 - I2013 == 0 155.778 382.825 0.407 1.0000
## N2016 - I2013 == 0 1123.630 382.825 2.935 0.0897 .
## I2015 - I2014 == 0 -7.532 273.189 -0.028 1.0000
## I2016 - I2014 == 0 1236.772 273.189 4.527 <0.01 ***
## N2011 - I2014 == 0 -22.665 297.496 -0.076 1.0000
## N2012 - I2014 == 0 -17.192 244.347 -0.070 1.0000
## N2013 - I2014 == 0 46.944 382.825 0.123 1.0000
## N2014 - I2014 == 0 -39.167 382.825 -0.102 1.0000
## N2015 - I2014 == 0 6.579 382.825 0.017 1.0000
## N2016 - I2014 == 0 974.431 382.825 2.545 0.2319
## I2016 - I2015 == 0 1244.304 284.382 4.375 <0.01 ***
## N2011 - I2015 == 0 -15.134 311.166 -0.049 1.0000
## N2012 - I2015 == 0 -9.660 258.791 -0.037 1.0000
## N2013 - I2015 == 0 54.475 392.315 0.139 1.0000
## N2014 - I2015 == 0 -31.636 392.315 -0.081 1.0000
## N2015 - I2015 == 0 14.111 392.315 0.036 1.0000
## N2016 - I2015 == 0 981.963 392.315 2.503 0.2535
## N2011 - I2016 == 0 -1259.438 311.166 -4.047 <0.01 **
## N2012 - I2016 == 0 -1253.964 258.791 -4.845 <0.01 ***
## N2013 - I2016 == 0 -1189.829 392.315 -3.033 0.0684 .
## N2014 - I2016 == 0 -1275.940 392.315 -3.252 0.0349 *
## N2015 - I2016 == 0 -1230.193 392.315 -3.136 0.0503 .
## N2016 - I2016 == 0 -262.341 392.315 -0.669 0.9996
## N2012 - N2011 == 0 5.473 278.935 0.020 1.0000
## N2013 - N2011 == 0 69.609 394.491 0.176 1.0000
## N2014 - N2011 == 0 -16.502 394.491 -0.042 1.0000
## N2015 - N2011 == 0 29.244 394.491 0.074 1.0000
## N2016 - N2011 == 0 997.096 394.491 2.528 0.2410
## N2013 - N2012 == 0 64.136 364.668 0.176 1.0000
## N2014 - N2012 == 0 -21.975 364.668 -0.060 1.0000
## N2015 - N2012 == 0 23.771 364.668 0.065 1.0000
## N2016 - N2012 == 0 991.623 364.668 2.719 0.1564
## N2014 - N2013 == 0 -86.111 449.647 -0.192 1.0000
## N2015 - N2013 == 0 -40.365 449.647 -0.090 1.0000
## N2016 - N2013 == 0 927.487 449.647 2.063 0.5379
## N2015 - N2014 == 0 45.746 449.647 0.102 1.0000
## N2016 - N2014 == 0 1013.598 449.647 2.254 0.4029
## N2016 - N2015 == 0 967.852 449.647 2.152 0.4741
## ---
## Signif. codes: 0 '***' 0.001 '**' 0.01 '*' 0.05 '.' 0.1 ' ' 1
## (Adjusted p values reported -- single-step method)

# plot(m1) # residuals show some heteroschedasticity, largely due to 1 outlier.

Significant differences in invertebrate density with Hydrobiids (p<0.0011); Impacted sites in 2016 were greater than Impacted sites in 2013, 2014,2015 (p<0.001) and non-impacted sites in 2011(p=0.002), 2012 (p<0.001), 2014 (p= 0.035), and marginally different from non-impacted sites in 2013 and 2015 (p=0.0684 and 0.0503).

m1<-lme(Richness.taxa2~Impact.Yr,random=~1|Site,data=damsub)
anova(m1) #

## numDF denDF F-value p-value
## (Intercept) 1 25 423.9375 <.0001
## Impact.Yr 9 25 1.7902 0.1206

# summary(glht(m1,linfct=mcp(Impact.Yr="Tukey")))
# plot(m1) # residuals OK

No significant differences in richness associated with Impact*year groupings.

If the Hydrobiids are included the richness increases in Impacted sites in 2016 compared to non-impacted 2011 sites, and marginally increases in Impactes sites in 2015.

m1<-lme(EPT.pct.noHyd~Impact.Yr,random=~1|Site,data=damsub)
anova(m1) #

## numDF denDF F-value p-value
## (Intercept) 1 25 366.1081 <.0001
## Impact.Yr 9 25 3.6034 0.0053

out<-glht(m1,linfct=mcp(Impact.Yr="Tukey"))
# plot(m1) # residuals OK

For EPT percent 2013 Impacted sites different from all other sites except non-impacted sites in 2011, 2013, & 2016. No other significant differences.

ffg$Imp.Yr<- factor(paste(ffg$Imp,ffg$Year))

m1<-lme(CG~Imp.Yr,random=~1|Site,data=droplevels(subset(ffg,Imp!="New"&Year>2010)))
anova(m1) #

## numDF denDF F-value p-value
## (Intercept) 1 25 36.53450 <.0001
## Imp.Yr 9 25 0.88307 0.553

#out<-glht(m1,linfct=mcp(Impact.Yr="Tukey"))
# plot(m1) # residuals OK

m1<-lme(CF~Imp.Yr,random=~1|Site,data=droplevels(subset(ffg,Imp!="New"&Year>2010)))
anova(m1) #

## numDF denDF F-value p-value
## (Intercept) 1 25 22.747428 0.0001
## Imp.Yr 9 25 0.764949 0.6488

#out<-glht(m1,linfct=mcp(Impact.Yr="Tukey"))
# plot(m1) # residuals OK

m1<-lme(PR~Imp.Yr,random=~1|Site,data=droplevels(subset(ffg,Imp!="New"&Year>2010)))
anova(m1) #

## numDF denDF F-value p-value
## (Intercept) 1 25 29.968450 <.0001
## Imp.Yr 9 25 1.669275 0.1497

#out<-glht(m1,linfct=mcp(Impact.Yr="Tukey"))
# plot(m1) # residuals OK

m1<-lme(SC.noHydro~Imp.Yr,random=~1|Site,data=droplevels(subset(ffg,Imp!="New"&Year>2010)))
anova(m1) #

## numDF denDF F-value p-value
## (Intercept) 1 25 16.60033 0.0004
## Imp.Yr 9 25 1.53199 0.1910

# out<-glht(m1,linfct=mcp(Impact.Yr="Tukey"))
# plot(m1) # residuals OK

No difference between Impact*Year for CG, CF, SC, or PR.

m1<-lme(SH~Imp.Yr,random=~1|Site,data=droplevels(subset(ffg,Imp!="New"&Year>2010)))
anova(m1) #

## numDF denDF F-value p-value
## (Intercept) 1 25 120.25452 <.0001
## Imp.Yr 9 25 17.78542 <.0001

out<-summary(glht(m1,linfct=mcp(Imp.Yr="Tukey")))

## Warning in RET$pfunction("adjusted", ...): Completion with error > abseps

## Warning in RET$pfunction("adjusted", ...): Completion with error > abseps

## Warning in RET$pfunction("adjusted", ...): Completion with error > abseps

## Warning in RET$pfunction("adjusted", ...): Completion with error > abseps

## Warning in RET$pfunction("adjusted", ...): Completion with error > abseps

## Warning in RET$pfunction("adjusted", ...): Completion with error > abseps

out

##
## Simultaneous Tests for General Linear Hypotheses
##
## Multiple Comparisons of Means: Tukey Contrasts
##
##
## Fit: lme.formula(fixed = SH ~ Imp.Yr, data = droplevels(subset(ffg,
## Imp != "New" & Year > 2010)), random = ~1 | Site)
##
## Linear Hypotheses:
## Estimate Std. Error z value Pr(>|z|)
## I 2014 - I 2013 == 0 -1.6667 2.0716 -0.805 0.9984
## I 2015 - I 2013 == 0 3.9000 2.1727 1.795 0.7256
## I 2016 - I 2013 == 0 20.1000 2.1727 9.251 <0.01 ***
## N 2011 - I 2013 == 0 -1.2500 2.3161 -0.540 0.9999
## N 2012 - I 2013 == 0 -2.0000 1.9378 -1.032 0.9894
## N 2013 - I 2013 == 0 0.5000 2.9297 0.171 1.0000
## N 2014 - I 2013 == 0 -3.0000 2.9297 -1.024 0.9900
## N 2015 - I 2013 == 0 8.5000 2.9297 2.901 0.0986 .
## N 2016 - I 2013 == 0 3.5000 2.9297 1.195 0.9711
## I 2015 - I 2014 == 0 5.5667 2.1727 2.562 0.2240
## I 2016 - I 2014 == 0 21.7667 2.1727 10.018 <0.01 ***
## N 2011 - I 2014 == 0 0.4167 2.3161 0.180 1.0000
## N 2012 - I 2014 == 0 -0.3333 1.9378 -0.172 1.0000
## N 2013 - I 2014 == 0 2.1667 2.9297 0.740 0.9992
## N 2014 - I 2014 == 0 -1.3333 2.9297 -0.455 1.0000
## N 2015 - I 2014 == 0 10.1667 2.9297 3.470 0.0170 *
## N 2016 - I 2014 == 0 5.1667 2.9297 1.764 0.7455
## I 2016 - I 2015 == 0 16.2000 2.2693 7.139 <0.01 ***
## N 2011 - I 2015 == 0 -5.1500 2.4070 -2.140 0.4835
## N 2012 - I 2015 == 0 -5.9000 2.0455 -2.884 0.1040
## N 2013 - I 2015 == 0 -3.4000 3.0020 -1.133 0.9798
## N 2014 - I 2015 == 0 -6.9000 3.0020 -2.298 0.3748
## N 2015 - I 2015 == 0 4.6000 3.0020 1.532 0.8715
## N 2016 - I 2015 == 0 -0.4000 3.0020 -0.133 1.0000
## N 2011 - I 2016 == 0 -21.3500 2.4070 -8.870 <0.01 ***
## N 2012 - I 2016 == 0 -22.1000 2.0455 -10.804 <0.01 ***
## N 2013 - I 2016 == 0 -19.6000 3.0020 -6.529 <0.01 ***
## N 2014 - I 2016 == 0 -23.1000 3.0020 -7.695 <0.01 ***
## N 2015 - I 2016 == 0 -11.6000 3.0020 -3.864 <0.01 **
## N 2016 - I 2016 == 0 -16.6000 3.0020 -5.530 <0.01 ***
## N 2012 - N 2011 == 0 -0.7500 2.1973 -0.341 1.0000
## N 2013 - N 2011 == 0 1.7500 3.1074 0.563 0.9999
## N 2014 - N 2011 == 0 -1.7500 3.1074 -0.563 0.9999
## N 2015 - N 2011 == 0 9.7500 3.1074 3.138 0.0503 .
## N 2016 - N 2011 == 0 4.7500 3.1074 1.529 0.8732
## N 2013 - N 2012 == 0 2.5000 2.8366 0.881 0.9967
## N 2014 - N 2012 == 0 -1.0000 2.8366 -0.353 1.0000
## N 2015 - N 2012 == 0 10.5000 2.8366 3.702 <0.01 **
## N 2016 - N 2012 == 0 5.5000 2.8366 1.939 0.6273
## N 2014 - N 2013 == 0 -3.5000 3.5881 -0.975 0.9930
## N 2015 - N 2013 == 0 8.0000 3.5881 2.230 0.4204
## N 2016 - N 2013 == 0 3.0000 3.5881 0.836 0.9978
## N 2015 - N 2014 == 0 11.5000 3.5881 3.205 0.0409 *
## N 2016 - N 2014 == 0 6.5000 3.5881 1.812 0.7148
## N 2016 - N 2015 == 0 -5.0000 3.5881 -1.393 0.9245
## ---
## Signif. codes: 0 '***' 0.001 '**' 0.01 '*' 0.05 '.' 0.1 ' ' 1
## (Adjusted p values reported -- single-step method)

# plot(m1) # residuals OK

Strong differences associated with shredders (p<0.0001). Impacted sites in 2016 had greater abundance of shredders than other site*years. Non-impacted sites in 2015 were higher than non-impacted sites in 2012 or 2014.

**Conclusions** We can draw 3 conclusions here:

1. No overall differences between sites possilby exposed to flooding created by the removal of the dam.
2. Lower %EPT in 2013 in impacted sites compared to all most other groups (Impacted vs non for each year)with the exception of 2011 sites (all non-impacted in 2011 and non-impacted sites in 2013.

### Linear model analysis of FFG data

All FFG groups expcept parasite. CG = Collector/Gatherer; CF = Collector/Filterer; SC = Scraper; SC.noH = Scraper (no Hydrobiids); SH = Shredder; PR = Predator; PA = Parasite

#### Site*Year Impact classes and FFG relative abundance

ffg$Tot.noH<-rowSums(ffg[,c(3:4,6:8)])
ffg$CG_rel<-ffg$CG/ffg$Tot.noH
ffg$CF_rel<-ffg$CF/ffg$Tot.noH
ffg$SC.noHydro_rel<-ffg$SC.noHydro/ffg$Tot.noH
ffg$SH_rel<-ffg$SH/ffg$Tot.noH
ffg$PR_rel<-ffg$PR/ffg$Tot.noH

m1<-lme(CG_rel~Impact,random=~1|Site/Year,data=ffg)
anova(m1)

## numDF denDF F-value p-value
## (Intercept) 1 43 280.37894 <.0001
## Impact 3 43 5.86135 0.0019

summary(glht(m1,linfct=mcp(Impact="Tukey")))

##
## Simultaneous Tests for General Linear Hypotheses
##
## Multiple Comparisons of Means: Tukey Contrasts
##
##
## Fit: lme.formula(fixed = CG_rel ~ Impact, data = ffg, random = ~1 |
## Site/Year)
##
## Linear Hypotheses:
## Estimate Std. Error z value Pr(>|z|)
## Downstream-after - Upstream == 0 -0.008739 0.083505 -0.105 0.9996
## Downstream-before - Upstream == 0 -0.137057 0.088004 -1.557 0.3925
## New - Upstream == 0 0.210149 0.096824 2.170 0.1256
## Downstream-before - Downstream-after == 0 -0.128318 0.052310 -2.453 0.0642
## New - Downstream-after == 0 0.218888 0.080305 2.726 0.0305
## New - Downstream-before == 0 0.347206 0.084975 4.086 <0.001
##
## Downstream-after - Upstream == 0
## Downstream-before - Upstream == 0
## New - Upstream == 0
## Downstream-before - Downstream-after == 0 .
## New - Downstream-after == 0 *
## New - Downstream-before == 0 ***
## ---
## Signif. codes: 0 '***' 0.001 '**' 0.01 '*' 0.05 '.' 0.1 ' ' 1
## (Adjusted p values reported -- single-step method)

# plot(m1) # unexplained pattern in residuals but not heteroschedatic

# m12<-lme(CG_rel~Impact2,random=~1|Site/Year,data=ffg)
# anova(m12)
# summary(glht(m12,linfct=mcp(Impact2="Tukey")))

Strong differences in relative abundance of ffg=CG, ‘new’ differences from downstream-beofre and -after, but not from upstream. Pattern holds, but weaker if upstream separted into ‘before and after’

m1<-lme(CF_rel~Impact,random=~1|Site/Year,data=ffg)
anova(m1)

## numDF denDF F-value p-value
## (Intercept) 1 43 97.13611 <.0001
## Impact 3 43 1.63911 0.1944

summary(glht(m1,linfct=mcp(Impact="Tukey")))

##
## Simultaneous Tests for General Linear Hypotheses
##
## Multiple Comparisons of Means: Tukey Contrasts
##
##
## Fit: lme.formula(fixed = CF_rel ~ Impact, data = ffg, random = ~1 |
## Site/Year)
##
## Linear Hypotheses:
## Estimate Std. Error z value Pr(>|z|)
## Downstream-after - Upstream == 0 0.03184 0.03316 0.960 0.770
## Downstream-before - Upstream == 0 0.05453 0.03635 1.500 0.434
## New - Upstream == 0 -0.02422 0.04075 -0.594 0.933
## Downstream-before - Downstream-after == 0 0.02269 0.03159 0.718 0.889
## New - Downstream-after == 0 -0.05607 0.03656 -1.533 0.414
## New - Downstream-before == 0 -0.07876 0.03948 -1.995 0.188
## (Adjusted p values reported -- single-step method)

# plot(m1) # unexplained pattern in residuals but not heteroschedatic

# m12<-lme(CF_rel~Impact2,random=~1|Site/Year,data=ffg)
# anova(m12)
# summary(glht(m12,linfct=mcp(Impact2="Tukey")))

No differences for CF.

m1<-lme(SC.noHydro_rel~Impact,random=~1|Site/Year,data=ffg)
anova(m1)

## numDF denDF F-value p-value
## (Intercept) 1 43 81.04654 <.0001
## Impact 3 43 6.41476 0.0011

summary(glht(m1,linfct=mcp(Impact="Tukey")))

##
## Simultaneous Tests for General Linear Hypotheses
##
## Multiple Comparisons of Means: Tukey Contrasts
##
##
## Fit: lme.formula(fixed = SC.noHydro_rel ~ Impact, data = ffg, random = ~1 |
## Site/Year)
##
## Linear Hypotheses:
## Estimate Std. Error z value Pr(>|z|)
## Downstream-after - Upstream == 0 -0.003733 0.064697 -0.058 0.99993
## Downstream-before - Upstream == 0 0.130139 0.067923 1.916 0.21204
## New - Upstream == 0 -0.118351 0.074769 -1.583 0.37661
## Downstream-before - Downstream-after == 0 0.133872 0.038753 3.454 0.00273
## New - Downstream-after == 0 -0.114618 0.061673 -1.858 0.23632
## New - Downstream-before == 0 -0.248490 0.065049 -3.820 < 0.001
##
## Downstream-after - Upstream == 0
## Downstream-before - Upstream == 0
## New - Upstream == 0
## Downstream-before - Downstream-after == 0 **
## New - Downstream-after == 0
## New - Downstream-before == 0 ***
## ---
## Signif. codes: 0 '***' 0.001 '**' 0.01 '*' 0.05 '.' 0.1 ' ' 1
## (Adjusted p values reported -- single-step method)

# plot(m1) # unexplained pattern in residuals but not heteroschedatic

# m12<-lme(SC.noHydro_rel~Impact2,random=~1|Site/Year,data=ffg)
# anova(m12)
# summary(glht(m12,linfct=mcp(Impact2="Tukey")))

Strong differences in relative abundance of SC(w/o hydrobiids). Downstream-before different from downstream-after and New

m1<-lme(SH_rel~Impact,random=~1|Site/Year,data=ffg)
anova(m1)

## numDF denDF F-value p-value
## (Intercept) 1 43 56.11866 <.0001
## Impact 3 43 1.15828 0.3367

summary(glht(m1,linfct=mcp(Impact="Tukey")))

##
## Simultaneous Tests for General Linear Hypotheses
##
## Multiple Comparisons of Means: Tukey Contrasts
##
##
## Fit: lme.formula(fixed = SH_rel ~ Impact, data = ffg, random = ~1 |
## Site/Year)
##
## Linear Hypotheses:
## Estimate Std. Error z value Pr(>|z|)
## Downstream-after - Upstream == 0 0.009882 0.014907 0.663 0.910
## Downstream-before - Upstream == 0 -0.016178 0.016341 -0.990 0.753
## New - Upstream == 0 -0.005125 0.018317 -0.280 0.992
## Downstream-before - Downstream-after == 0 -0.026060 0.014201 -1.835 0.254
## New - Downstream-after == 0 -0.015007 0.016436 -0.913 0.796
## New - Downstream-before == 0 0.011053 0.017747 0.623 0.924
## (Adjusted p values reported -- single-step method)

# plot(m1) # unexplained pattern in residuals but not heteroschedatic

# m12<-lme(SH_rel~Impact2,random=~1|Site/Year,data=ffg)
# anova(m12)
# summary(glht(m12,linfct=mcp(Impact2="Tukey")))

No significant differences in SH relative abundance

m1<-lme(PR_rel~Impact,random=~1|Site/Year,data=ffg)
anova(m1)

## numDF denDF F-value p-value
## (Intercept) 1 43 92.04741 <.0001
## Impact 3 43 1.10834 0.3562

summary(glht(m1,linfct=mcp(Impact="Tukey")))

##
## Simultaneous Tests for General Linear Hypotheses
##
## Multiple Comparisons of Means: Tukey Contrasts
##
##
## Fit: lme.formula(fixed = PR_rel ~ Impact, data = ffg, random = ~1 |
## Site/Year)
##
## Linear Hypotheses:
## Estimate Std. Error z value Pr(>|z|)
## Downstream-after - Upstream == 0 -0.028919 0.028639 -1.010 0.741
## Downstream-before - Upstream == 0 -0.036241 0.031296 -1.158 0.650
## New - Upstream == 0 -0.062450 0.034837 -1.793 0.274
## Downstream-before - Downstream-after == 0 -0.007322 0.026073 -0.281 0.992
## New - Downstream-after == 0 -0.033530 0.030901 -1.085 0.696
## New - Downstream-before == 0 -0.026209 0.033379 -0.785 0.860
## (Adjusted p values reported -- single-step method)

# plot(m1) # unexplained pattern in residuals but not heteroschedatic

# m12<-lme(PR_rel~Impact2,random=~1|Site/Year,data=ffg)
# anova(m12)
# summary(glht(m12,linfct=mcp(Impact2="Tukey")))

No significant differences in PR relative abundance

### Redo Figure 2

Key response parameters (Invert Density, Taxon Richness, and EPT%) overthe sites and years.


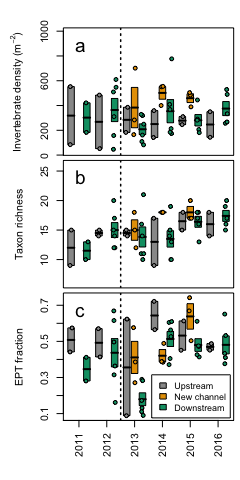


###### Figure 2. Distribution of key response parameters in upstream and downstream sites from 2011-2016. Rectangles show mean +/- SE and the mean divides each rectangle. The actual data values are shown. (Hydrobiid snails not included)

Note the increase in spread in the “Upstream” sites in 2013 - the site GHL was near enough to the impoundment that it may have been affected by the draining of the impoundment.

### Improve Taxa Specific Analysis

I think that we need to improve our taxa specific analysis and interpretation. In my thesis I ended up using indicator analysis to look at specific taxa associations to impact classes, and found it more intuitive than SIMPER percentages. It ranks and provides a P-value for taxa that are significant indicators of site categories. I suggest we use this as a follow up to the PERMANOVA/NMDS results, and could provide Eric the R code, or do it myself. (see methods in this draft for packages I suggest using). I think we could do indicator analysis for the site categories, and then also for year (just for the 5 downstream sites).

- Indicator species analsysis on PERMANOVA/NMDS results. - Data sent to Joel on 1/20/20

### Improve New Zealand Mud Snail analysis

The following tallies NZMS abundance by site and year, and calculates t-tests to compare each year with the prior year.

Using corrected density data

# library(readr)
# mudsnail <- read_csv("MudSnail Density Table.csv", col_types = cols(Impact = col_character()), comment = "#") # these values were calculated somewhere else (Excel maybe?) - the following recalculates these values from the data in R.
mudsnail2 <- aggregate(Hydrobiidae~Site+Year,sum,data=mv_fam)
mudsnail2$Hydrobiidae<-mudsnail2$Hydrobiidae/0.558
## now create rows for missing site*years
mudsnail2[58:72,]<-NA
mudsnail2[58:66,1]<-rep(c("BBU","BBM","BBL"),3)
mudsnail2[58:66,2]<-rep(c("2011","2012","2016"),each=3)
mudsnail2[67:69,1]<-c("14P","WYR","SUM")
mudsnail2[67:69,2]<-"2011"
mudsnail2[70:71,1]<-rep("LP",2)
mudsnail2[70:71,2]<-c("2015","2016")
mudsnail2[72,1:2]<-c("BBR","2011")
mudsnail2<-mudsnail2[-(1:6),] # remove 2008-2011
## sort by site and year.
mudsnail2<-mudsnail2[order(mudsnail2$Site,mudsnail2$Year),]
## unstack to create a table with columns for years
mudsnail2<-unstack(mudsnail2,Hydrobiidae~Year)
rownames(mudsnail2)<-sort(unique(mv_fam$Site))
mudsnail2<-mudsnail2[c(7,6,5,3,2,4,1,11,10,9,8),] #sort by physical location

apply(X=mudsnail2,MAR=2,FUN=mean,na.rm=T)

## X2011 X2012 X2013 X2014 X2015 X2016
## 0.00000 0.00000 34.05018 34.05018 75.80645 1186.63594

apply(X=mudsnail2,MAR=2,FUN=sd,na.rm=T)

## X2011 X2012 X2013 X2014 X2015 X2016
## 0.00000 0.00000 63.09160 43.76577 53.55095 959.63429

apply(X=mudsnail2,MAR=2,FUN=function(x)(sd(x,na.rm=T)/sqrt(sum(is.na(x)==FALSE))))

## X2011 X2012 X2013 X2014 X2015 X2016
## 0.00000 0.00000 19.02283 13.19588 16.93430 362.70767

out<-matrix(NA,nrow=3,ncol=5)
for (i in 2:6){
 z<-t.test(x=mudsnail2[[(i-1)]],y=mudsnail2[[i]])
 out[1,i-1]<-z$statistic
 out[2,i-1]<-z$parameter
 out[3,i-1]<-z$p.value
}
# dimnames(out)=list(c("t-value","df","p-value"),c("2012","2013","2014","2015","2016"))
# library(knitr)
# kable(signif(out,4))

#huge differences in sd make t-tests invalid

lmudsnail<-mudsnail2
lmudsnail<-log(lmudsnail+0.1)

apply(X=lmudsnail,MAR=2,FUN=mean,na.rm=T)

## X2011 X2012 X2013 X2014 X2015 X2016
## -2.302585 -2.302585 1.116451 1.760072 4.132900 6.889324

apply(X=lmudsnail,MAR=2,FUN=sd,na.rm=T)

## X2011 X2012 X2013 X2014 X2015 X2016
## 0.0000000 0.0000000 2.9762840 2.8112906 0.6394944 0.6021589

out<-matrix(NA,nrow=3,ncol=4)
for (i in 3:6){
 z<-t.test(x=lmudsnail[[(i-1)]],y=lmudsnail[[i]])
 out[1,i-2]<-z$statistic
 out[2,i-2]<-z$parameter
 out[3,i-2]<-z$p.value
}
dimnames(out)=list(c("t-value","df","p-value"),c("2013","2014","2015","2016"))
library(knitr)
kable(signif(out,4))

|  | 2013 | 2014 | 2015 | 2016 |
| --- | --- | --- | --- | --- |
| t-value | -3.810000 | -0.5214 | -2.72300 | -9.0540000 |
| df | 10.000000 | 19.9400 | 11.13000 | 13.5700000 |
| p-value | 0.003429 | 0.6078 | 0.01964 | 0.0000004 |

###. Test whether there is multivariate grouping associated with the Impact classes.

We can use MDS (Non-metric Multi Dimenisional Scaling) to visualise the data, and PERMANOVA to test whether the groups defined by impact are distinct from each other in multi-dimensional space.

NMDS and PERMANOVA are typically carried out on transformed data, but this leaves the question of which transformation is appropriate. We considered square-root, Wisconsin double standardization (on both raw and square-root transformed data), log(x+1) and fourth-root transformations.

## This is vegan 2.5-6


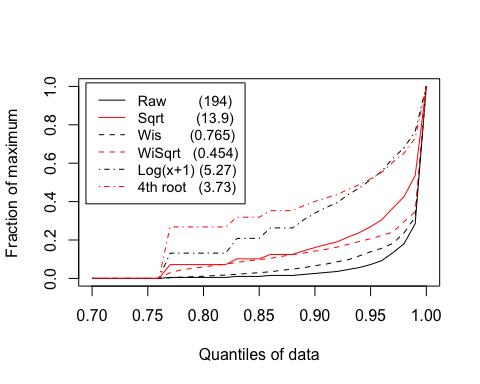


###### Relative effect of various transformations on the data. The data are transformed, and then standardized by the maximum value. The quantiles 0.7-1.0 are plotted here, and the maximum abundance for each transformation in listed in the legend.

This figure shows that the Wisconsin double standardization is the closest to a linear scaling of the raw data, while the fourth root transformation has the strongest boost of the lower abundances, with log(x+1) transformation giving similar results for top 10%, but only about half as much increase to abundances in the 75-85 percentiles. Square root and Wisconsin standardization of square root transformed data are quite similar, and generally provide a smaller boost for the lower abundances. Based on this analysis, it seems Wisconsin standardization will emphasize the abundant species the most, while fourth root will emphasize the less abundant species more than the other transformations (least abundant species only ~35% of the most abundant species).

### NMDS Figures

##### Figure 3

The results here are quite similar for k=3,4, or 5.

# k=3, 4th root transformation
mds1<-metaMDS(comm=mv_fam.taxa[-(1:6),-30]^0.25,k=3,trymax=50,autotransform = FALSE) #-1:6 to drop 08-10, -30 to omit Hydrobiidae

## Run 0 stress 0.1710489
## Run 1 stress 0.1708604
## ... New best solution
## ... Procrustes: rmse 0.01608636 max resid 0.08192273
## Run 2 stress 0.1738183
## Run 3 stress 0.1728434
## Run 4 stress 0.1758644
## Run 5 stress 0.1710575
## ... Procrustes: rmse 0.01647004 max resid 0.08540378
## Run 6 stress 0.1743626
## Run 7 stress 0.1774352
## Run 8 stress 0.170851
## ... New best solution
## ... Procrustes: rmse 0.005104097 max resid 0.02520734
## Run 9 stress 0.1767222
## Run 10 stress 0.1724684
## Run 11 stress 0.1738197
## Run 12 stress 0.1743729
## Run 13 stress 0.1774574
## Run 14 stress 0.1708493
## ... New best solution
## ... Procrustes: rmse 0.0007760441 max resid 0.003458711
## ... Similar to previous best
## Run 15 stress 0.1727795
## Run 16 stress 0.1710519
## ... Procrustes: rmse 0.01501502 max resid 0.06712642
## Run 17 stress 0.1708542
## ... Procrustes: rmse 0.001360422 max resid 0.005459583
## ... Similar to previous best
## Run 18 stress 0.1733295
## Run 19 stress 0.1758632
## Run 20 stress 0.1716264
## *** Solution reached

taxafit<-envfit(mds1,env=mv_fam.taxa[-(1:6),-30]^0.25 ,permutations=999)

stressplot(mds1)


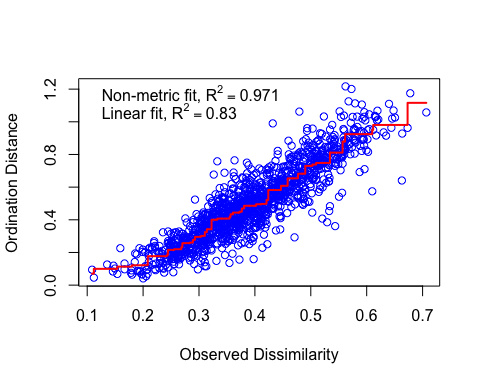


# Non-metric fit R^2 = 0.971; Linear fit, R^2 = 0.83

## quartz(width=7,height=5)

# all.equal(row.names(mds1$points),mv_fam$Site.Yr) # TRUE, so we can use groupd data from mv_fam for plotting mds1 results
# export to pdf at 5x7" for conversion to eps
par(mar=c(0,0,0,0),oma=c(4,4,0,1), family="Arial")
layout(matrix(c(3,1,3,2),ncol=2),heights=c(0.2,1))
imp<-mv_fam$Impact[-(1:6)]
sym<-c(24,25,25,21);ltys<-c(2,3,3,2)

 cols<-c("#000000","#009E73","#0072B2","#E69F00") # https://jfly.uni-koeln.de/color/
# cols<-c("#D81B60","#1E88E5","#FFC107","#004D40") # from https://davidmathlogic.com/colorblind/
ordiplot(mds1, type = "n",choices = c(1,2), family="Arial", cex=0.9,cex.lab=0.9,cex.axis=0.9,xaxs="i",yaxs="i")
mtext(side=c(1,2),text = c("NMDS1","NMDS2"),line=2.5)
orditorp(mds1,choices=c(1,2), display="species",air=0.01,col = "grey55",cex=0.45) ## add taxa
with(mv_fam[-(1:6),], ordihull(mds1, Impact, scaling = "symmetric", label = FALSE,col=cols,lty=ltys,choices=c(1,2))) ## ordination hulls
text(x=mds1$points[,1],y=mds1$points[,2],labels = mv_fam$Year[-(1:6)]-2000, cex=0.6, col=cols[as.numeric(imp)]) ## add sites

ordiplot(mds1, type = "n",choices = c(3,2), family="Arial", cex=0.9,cex.lab=0.9,cex.axis=0.9, yaxt="n",xaxs="i",yaxs="i")
mtext(side=1,text = "NMDS3",line=2.5)
orditorp(mds1,choices=c(3,2),display="species",air=0.01,col = "grey55",cex=0.45) ## add taxa
with(mv_fam[-(1:6),], ordihull(mds1, Impact, scaling = "symmetric", label = FALSE,col=cols,lty=ltys,choices=c(3,2)))
text(x=mds1$points[,3],y=mds1$points[,2],labels = mv_fam$Year[-(1:6)]-2000, cex=0.6, col=cols[as.numeric(imp)])

plot.new()
lord<-c(1,2,4,3)
legend("center",xpd=NA,lty=ltys[lord],col=cols[lord],pch=4,legend = c("Upstream","Downstream-before","New channel","Downstream-after"),ncol=2) # legend = levels(imp)[lord]


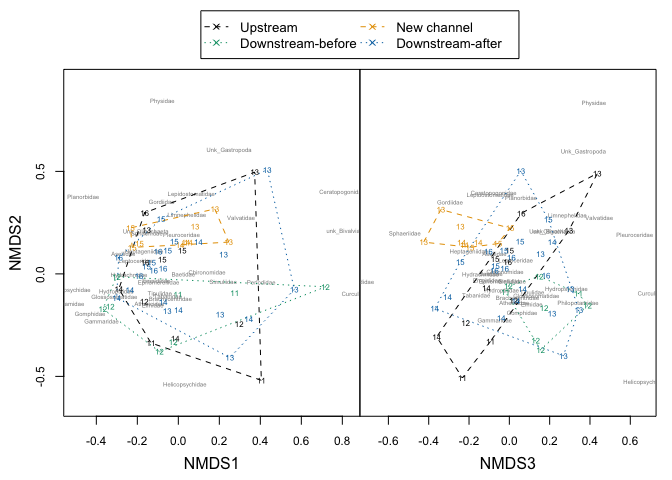


If we want to know which taxa are aligned with which ordination axes, we can look for ratio of axis 1 value to the pythagorean distance of axis 3 &axis 2 value.

# which(abs(mds1$species[,1])>0.67) # large axis 1 values
# this just means far from the origin

# tol=3
# # which(abs(mds1$species[,1]/mds1$species[,2])>tol & abs(mds1$species[,1]/mds1$species[,3])>tol)
# this gets all with tangent < 1/tol

# better to calculate the tangent for each taxa wrt each axis, and return the 4 taxa with smallest (absolute value of) tangent (nearest to parallel) for each axis.

taxaDist<-data.frame(mds1$species)
taxaDist$Axis1<-sqrt(taxaDist[,2]^2+taxaDist[,3]^2)/taxaDist[,1]
taxaDist$Axis2<-sqrt(taxaDist[,1]^2+taxaDist[,3]^2)/taxaDist[,2]
taxaDist$Axis3<-sqrt(taxaDist[,2]^2+taxaDist[,1]^2)/taxaDist[,3]

axisTaxa<-data.frame(
 row.names(taxaDist)[order(abs(taxaDist$Axis1))][1:4],
 taxaDist$Axis1[order(abs(taxaDist$Axis1))][1:4],
 row.names(taxaDist)[order(abs(taxaDist$Axis2))][1:4],
 taxaDist$Axis2[order(abs(taxaDist$Axis2))][1:4],
 row.names(taxaDist)[order(abs(taxaDist$Axis3))][1:4],
 taxaDist$Axis3[order(abs(taxaDist$Axis3))][1:4])
names(axisTaxa)<-c("Axis1 Taxa","Tan Axis1", "Axis2 Taxa","Tan Axis 2","Axis3 Taxa","Tan Axis3")
library(knitr,quietly = TRUE)
kable(axisTaxa)

| Axis1 Taxa | Tan Axis1 | Axis2 Taxa | Tan Axis 2 | Axis3 Taxa | Tan Axis3 |
| --- | --- | --- | --- | --- | --- |
| Chironomidae | 0.1610429 | Lepidostomatidae | 0.2931315 | Baetidae | -0.2742472 |
| Simuliidae | 0.1829395 | Brachycentridae | -0.4575091 | Pleuroceridae | 0.3094153 |
| Hydroptilidae | -0.3145470 | Physidae | 0.5065509 | Sphaeriidae | -0.4813910 |
| Leptoceridae | -0.3349504 | Unk_Gastropoda | 0.7343112 | Perlodidae | -0.5485068 |

If we want to know which taxa are aligned with which ordination axes, we can look for ratio of axis 1 value to the pythagorean distance of axis 3 &axis 2 value, and same for the others. A small tangent means the angle between that taxa and that axis is small.

taxaDist<-data.frame(mds1$species)
taxaDist$Axis1<-sqrt(taxaDist[,2]^2+taxaDist[,3]^2)/taxaDist[,1]
taxaDist$Axis2<-sqrt(taxaDist[,1]^2+taxaDist[,3]^2)/taxaDist[,2]
taxaDist$Axis3<-sqrt(taxaDist[,2]^2+taxaDist[,1]^2)/taxaDist[,3]

axisTaxa<-data.frame(
 row.names(taxaDist)[order(abs(taxaDist$Axis1))][1:4],
 taxaDist$Axis1[order(abs(taxaDist$Axis1))][1:4],
 row.names(taxaDist)[order(abs(taxaDist$Axis2))][1:4],
 taxaDist$Axis2[order(abs(taxaDist$Axis2))][1:4],
 row.names(taxaDist)[order(abs(taxaDist$Axis3))][1:4],
 taxaDist$Axis3[order(abs(taxaDist$Axis3))][1:4])
names(axisTaxa)<-c("Axis1 Taxa","Tan Axis1", "Axis2 Taxa","Tan Axis 2","Axis3 Taxa","Tan Axis3")
library(knitr,quietly = TRUE)
kable(axisTaxa)

| Axis1 Taxa | Tan Axis1 | Axis2 Taxa | Tan Axis 2 | Axis3 Taxa | Tan Axis3 |
| --- | --- | --- | --- | --- | --- |
| Chironomidae | 0.1610429 | Lepidostomatidae | 0.2931315 | Baetidae | -0.2742472 |
| Simuliidae | 0.1829395 | Brachycentridae | -0.4575091 | Pleuroceridae | 0.3094153 |
| Hydroptilidae | -0.3145470 | Physidae | 0.5065509 | Sphaeriidae | -0.4813910 |
| Leptoceridae | -0.3349504 | Unk_Gastropoda | 0.7343112 | Perlodidae | -0.5485068 |

### PERMANOVA

We need to check the assumption of similar dispersion among the groups, as the assumption of similar within-group dispersion is important for PERMANOVA. Also note that NMDS and PERMANOVA are carried out on fourth-root transformed data.

bd<-betadisper(vegdist(mv_fam.taxa[-(1:6),-30]^0.25),mv_fam$Impact[-(1:6)])
par(mfrow=c(1,1),mar=c(4.2,7.2,1,1),oma=rep(0,4), family="Arial")
boxplot(bd,col="grey",yaxt="n",horizontal=TRUE,xlab="Distance to centroid",ylab="")
# axis(side=2,cex.axis=0.8,labels = levels(droplevels(mv_fam$Impact[-new.excl])),at=1:4,las=1)
axis(side=2,cex.axis=0.8,labels = levels(mv_fam$Impact), at=1:4,las=1)


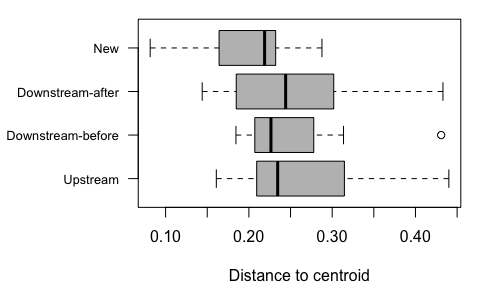


bd.pt<-permutest(bd,permutations=999,pairwise=TRUE)

###### Multivariate dispersion of community data for the three site impact classes for 2013-2015.

# anova(bd) # p = 0.116
# permutest(bd,permutations=999) #p=0.102
bd.pt

##
## Permutation test for homogeneity of multivariate dispersions
## Permutation: free
## Number of permutations: 999
##
## Response: Distances
## Df Sum Sq Mean Sq F N.Perm Pr(>F)
## Groups 3 0.033111 0.0110370 1.6455 999 0.203
## Residuals 47 0.315241 0.0067073
##
## Pairwise comparisons:
## (Observed p-value below diagonal, permuted p-value above diagonal)
## Upstream Downstream-before Downstream-after New
## Upstream 0.716000 0.602000 0.050
## Downstream-before 0.707869 0.961000 0.114
## Downstream-after 0.596156 0.966426 0.075
## New 0.046601 0.114723 0.068101

# permutest(bd,permutations=999,pairwise=TRUE)
# new is different from the others.
# permutest(bd2,permutations=999,pairwise=TRUE)
# this is true regardless of whether we use fourth root or wisconisin transformation

We have a problem with the “New” site*years. They are fewer in number and less dipsersed than the other site*years. This is true for both fourth-root transformation and wisconsin standardization. There are 2 possible ways to deal with this:
1) Drop them from the analysis. This could be justified because there is no “pre-removal” data from these sites.
2) Combine all the site*years that are not downstream of a dam and compare with the “Downstream-before” site*years.

bd3<-betadisper(vegdist(mv_fam.taxa2[-(1:6),-28]^0.25),mv_fam$Impact[-c(1:6,which(mv_fam$Impact=="New"))]) # run w/o "New", -28 to rm "Hydrobiidae"
cat("Dispersion of site classes excluding 'New' sites\n")

## Dispersion of site classes excluding 'New' sites

out<-permutest(bd3,permutations=999) #p=0.852
out

##
## Permutation test for homogeneity of multivariate dispersions
## Permutation: free
## Number of permutations: 999
##
## Response: Distances
## Df Sum Sq Mean Sq F N.Perm Pr(>F)
## Groups 2 0.002055 0.0010274 0.1431 999 0.878
## Residuals 39 0.279985 0.0071791

# permutest(bd3,permutations=999,pairwise=TRUE)

Impact2<-mv_fam$Impact
Impact2[which(Impact2%in%c("New","Upstream","Downstream-after"))]<-"Upstream"
Impact2<-droplevels(Impact2)
levels(Impact2)<-c("No","Yes") # downstream of a dam
betadisper(vegdist(mv_fam.taxa[-(1:6),-30]^0.25),Impact2[-(1:6)])

##
## Homogeneity of multivariate dispersions
##
## Call: betadisper(d = vegdist(mv_fam.taxa[-(1:6), -30]^0.25), group =
## Impact2[-(1:6)])
##
## No. of Positive Eigenvalues: 29
## No. of Negative Eigenvalues: 21
##
## Average distance to median:
## No Yes
## 0.2648 0.2548
##
## Eigenvalues for PCoA axes:
## (Showing 8 of 50 eigenvalues)
## PCoA1 PCoA2 PCoA3 PCoA4 PCoA5 PCoA6 PCoA7 PCoA8
## 0.7888 0.6964 0.4648 0.4478 0.3837 0.3114 0.2644 0.2106

cat("Dispersion of site classes grouped by being downstream of an intact dam\n")

## Dispersion of site classes grouped by being downstream of an intact dam

anova(betadisper(vegdist(mv_fam.taxa[,-30]^0.25),Impact2)) # p=0.45

## Analysis of Variance Table
##
## Response: Distances
## Df Sum Sq Mean Sq F value Pr(>F)
## Groups 1 0.00489 0.0048904 0.5681 0.4542
## Residuals 55 0.47348 0.0086086

Either of these approaches meet the assumption of similar dispersion, but approach 1 makes the most sense, as there is a good reason to exclude the “New” sites, and good reason to want to separate the upsteam site. Replotting the data also shows that (at least in the first 3 axes of our MDS) the dispersion appears similar and that the downstream-before group really does separate from the others.

Now we will fit a PERMANOVA to fourth-root transformed community data, using Bray-Curtis distance, and 10^6^ permutations for the p-value. Note that the Hydrobiid snails are not included here.

m1<-adonis(mv_fam.taxa2[-(1:6),-28]^0.25 ~ Impact, data=mv_fam[-c(1:6,which(mv_fam$Impact=="New")),], permutations=99999 ,method="bray")
m1 # significant difference

##
## Call:
## adonis(formula = mv_fam.taxa2[-(1:6), -28]^0.25 ~ Impact, data = mv_fam[-c(1:6, which(mv_fam$Impact == "New")), ], permutations = 99999, method = "bray")
##
## Permutation: free
## Number of permutations: 99999
##
## Terms added sequentially (first to last)
##
## Df SumsOfSqs MeanSqs F.Model R2 Pr(>F)
## Impact 2 0.4066 0.203282 2.6566 0.1199 3e-04 ***
## Residuals 39 2.9842 0.076519 0.8801
## Total 41 3.3908 1.0000
## ---
## Signif. codes: 0 '***' 0.001 '**' 0.01 '*' 0.05 '.' 0.1 ' ' 1

We have at least one highly significant difference. Likely the difference is the difference we spotted visually, but we need to check using multiple comparisons to be sure. We can carry out the same analysis on all three pair comparisons, protecting against inflation of error rate by using a bonferroni correction.

## using pairwise.adonis()
# devtools::install_github("pmartinezarbizu/pairwiseAdonis/pairwiseAdonis")
library(pairwiseAdonis)

## Loading required package: cluster

out<-pairwise.adonis(x=mv_fam.taxa2[-(1:6),-28]^0.25,factors = mv_fam$Impact[-c(1:6,which(mv_fam$Impact=="New"))],perm=9999)
out[,1]<-gsub(x = out$pairs,pattern = "stream",replacement = "",fixed=TRUE)
format(out,digits=6)

## pairs Df SumsOfSqs F.Model R2 p.value p.adjusted
## 1 Up vs Down-before 1 0.246024 2.98274 0.1421522 0.0011 0.0033
## 2 Up vs Down-after 1 0.166528 2.19756 0.0642608 0.0171 0.0513
## 3 Down-before vs Down-after 1 0.216442 2.94356 0.0951266 0.0036 0.0108
## sig
## 1 *
## 2
## 3 .

# pairwise.adonis(x=wisconsin(mv_fam.taxa2[,-28]),factors = mv_fam$Impact[-which(mv_fam$Impact=="New")],perm=9999)

## Include Hydrobiidae
# pairwise.adonis(x=mv_fam.taxa2[-(1:6),]^0.25,factors = mv_fam$Impact[-c(1:6,which(mv_fam$Impact=="New"))],perm=9999)

# library(pairwiseAdonis)
# library(vegan)
# want to confirm robustness of result to: 1) removal of taxa<=5, 2) transformation used 3) removal of 2008-2010
 # mv_fam 2 created in chunk "prep-mv-fam-abundance-data"

# 1) all taxa, 4th root ----
m2<-adonis(mv_fam2[-which(mv_fam$Impact=="New"),-57]^0.25 ~ Impact, data=mv_fam[-which(mv_fam$Impact=="New"),], permutations=99999 ,method="bray")
m2 # p <0.00001, 4th root, all taxa, (no hydrobiidae)
pairwise.adonis(mv_fam2[-which(mv_fam$Impact=="New"),-57]^0.25,factors = mv_fam$Impact[-which(mv_fam$Impact=="New")],perm=9999)
# D-b different from D-a and U (p<0.0005), D-a & U not diff (p=0.06)

# 1) All taxa,Wisconsin transformation ----
m2<-adonis(wisconsin(mv_fam2[-which(mv_fam$Impact=="New"),-57]) ~ Impact, data=mv_fam[-which(mv_fam$Impact=="New"),], permutations=99999 ,method="bray")
m2 # p <0.00021

pairwise.adonis(wisconsin(mv_fam2[-which(mv_fam$Impact=="New"),-57]),factors = mv_fam$Impact[-which(mv_fam$Impact=="New")],perm=9999)

# 1) All taxa,Hydrobiids included ----
m2<-adonis(mv_fam2[-which(mv_fam$Impact=="New"),]^0.25 ~ Impact, data=mv_fam[-which(mv_fam$Impact=="New"),], permutations=99999 ,method="bray")
m2 # p <0.00001

pairwise.adonis(mv_fam2[-which(mv_fam$Impact=="New"),]^0.25,factors = mv_fam$Impact[-which(mv_fam$Impact=="New")],perm=9999)

# 1) all taxa, drop 2008-2010 4th root, no hydrobiids ----
m2<-adonis(mv_fam2[-c(1:6,which(mv_fam$Impact=="New")),-57]^0.25 ~ Impact, data=mv_fam[-c(1:6,which(mv_fam$Impact=="New")),], permutations=99999 ,method="bray")
m2 # p <0.00018,
pairwise.adonis(mv_fam2[-c(1:6,which(mv_fam$Impact=="New")),-57]^0.25,factors = mv_fam$Impact[-c(1:6,which(mv_fam$Impact=="New"))],perm=9999)
# D-b different from D-a (p=0.0035) and U (p<0.008), D-a & U not diff (p=0.057)

## The main findings don't change when we use all the taxa, regardless of tranformation of presence of hydrobiids (though the marginally signficiant difference between upstream and down-after disapears), or inclusion of years 2008-2010

# 2) non-rare taxa, 4th root, all years ----
m2<-adonis(mv_fam.taxa[-which(mv_fam$Impact=="New"),-57]^0.25 ~ Impact, data=mv_fam[-which(mv_fam$Impact=="New"),], permutations=99999 ,method="bray")
m2 # p <0.00001, 4th root, all taxa, (no hydrobiidae)
pairwise.adonis(mv_fam.taxa[-which(mv_fam$Impact=="New"),-57]^0.25,factors = mv_fam$Impact[-which(mv_fam$Impact=="New")],perm=9999)
# D-b different from D-a and U (p<0.0005), D-a & U not diff (p=0.15)

# 2) non-rare taxa, 4th root, drop 2008-10 ----
m2<-adonis(mv_fam.taxa[-c(1:6,which(mv_fam$Impact=="New")),-57]^0.25 ~ Impact, data=mv_fam[-c(1:6,which(mv_fam$Impact=="New")),], permutations=99999 ,method="bray")
m2 # p <0.00023, 4th root, all taxa, (no hydrobiidae)
pairwise.adonis(mv_fam.taxa[-c(1:6,which(mv_fam$Impact=="New")),-57]^0.25,factors = mv_fam$Impact[-c(1:6,which(mv_fam$Impact=="New"))],perm=9999)
# D-b different from D-a and U (p<0.0035), D-a & U not diff (p=0.16)

# 2) non-rare taxa, 4th root, drop 2008-10, include Hydrobiidae ----
m2<-adonis(mv_fam.taxa[-c(1:6,which(mv_fam$Impact=="New")),]^0.25 ~ Impact, data=mv_fam[-c(1:6,which(mv_fam$Impact=="New")),], permutations=99999 ,method="bray")
m2 # p <0.00009, 4th root, common taxa+Hydrobiidae,
pairwise.adonis(mv_fam.taxa[-c(1:6,which(mv_fam$Impact=="New")),]^0.25,factors = mv_fam$Impact[-c(1:6,which(mv_fam$Impact=="New"))],perm=9999)
# D-b different from D-a and U (p<0.003), D-a & U not diff (p=0.15)

# 2) non-rare taxa,Wisconsin transformation, all years ----
m2<-adonis(wisconsin(mv_fam.taxa[-which(mv_fam$Impact=="New"),-57]) ~ Impact, data=mv_fam[-which(mv_fam$Impact=="New"),], permutations=99999 ,method="bray")
m2 # p <0.00017

pairwise.adonis(wisconsin(mv_fam2[-which(mv_fam$Impact=="New"),-57]),factors = mv_fam$Impact[-which(mv_fam$Impact=="New")],perm=9999)
# D-d still different from both U and Da (P<0.0013), Da, U not diff.

# 2) non-rare taxa,Wisconsin transformation, drop 2008-10 ----
m2<-adonis(wisconsin(mv_fam.taxa[-c(1:6,which(mv_fam$Impact=="New")),-57]) ~ Impact, data=mv_fam[-c(1:6,which(mv_fam$Impact=="New")),], permutations=99999 ,method="bray")
m2 # p <0.00053

pairwise.adonis(wisconsin(mv_fam2[-c(1:6,which(mv_fam$Impact=="New")),-57]),factors = mv_fam$Impact[-c(1:6,which(mv_fam$Impact=="New"))],perm=9999)
# D-d still different from both U and Da (P<0.0033), Da, U not diff (p=0.75).


# Whether rare taxa are inlcuded or not does not change results
# Whether 2008-2010 data are included does not change results
# Whether we use 4th root or Wisconsin transformation does not change results
# Whether we include Hydrobbidae or not does not change the results

#### Analsysis of Upstream Sites

We should verify whether upstream sites are altered by dam removal, by comparing these sites before and after.

m1<-adonis(mv_fam.taxa[-(1:6),-30]^0.25 ~ mv_fam$Impact2[-c(1:6)], permutations=99999 ,method="bray") #data=mv_fam[-c(1:6),],
m1 # significant difference

##
## Call:
## adonis(formula = mv_fam.taxa[-(1:6), -30]^0.25 ~ mv_fam$Impact2[-c(1:6)], permutations = 99999, method = "bray")
##
## Permutation: free
## Number of permutations: 99999
##
## Terms added sequentially (first to last)
##
## Df SumsOfSqs MeanSqs F.Model R2 Pr(>F)
## mv_fam$Impact2[-c(1:6)] 4 0.9125 0.228122 3.2359 0.21959 1e-05 ***
## Residuals 46 3.2429 0.070498 0.78041
## Total 50 4.1554 1.00000
## ---
## Signif. codes: 0 '***' 0.001 '**' 0.01 '*' 0.05 '.' 0.1 ' ' 1

out<-pairwise.adonis(x=mv_fam.taxa[-(1:6),-30]^0.25,factors = mv_fam$Impact2[-c(1:6)],perm=9999)
out[,1]<-gsub(x = out$pairs,pattern = "stream",replacement = "",fixed=TRUE)
format(out,digits=5)

## pairs Df SumsOfSqs F.Model R2 p.value p.adjusted
## 1 Up-before vs Down-before 1 0.19557 2.4766 0.198498 0.0200 0.200
## 2 Up-before vs Up-after 1 0.17171 2.2200 0.181672 0.0280 0.280
## 3 Up-before vs New 1 0.29241 5.3608 0.327662 0.0013 0.013
## 4 Up-before vs Down-after 1 0.21512 2.9154 0.108317 0.0064 0.064
## 5 Down-before vs Up-after 1 0.24792 3.1485 0.183601 0.0002 0.002
## 6 Down-before vs New 1 0.40997 6.6199 0.306196 0.0003 0.003
## 7 Down-before vs Down-after 1 0.21946 2.9264 0.094624 0.0024 0.024
## 8 Up-after vs New 1 0.21640 3.5565 0.191657 0.0015 0.015
## 9 Up-after vs Down-after 1 0.12823 1.7233 0.057977 0.0802 0.802
## 10 New vs Down-after 1 0.24804 3.7657 0.114929 0.0002 0.002
## sig
## 1
## 2
## 3 .
## 4
## 5 *
## 6 *
## 7 .
## 8 .
## 9
## 10 *

# at alpha=0.01: D-b different from U-a & N. D-a different from N
# at alpha=0.05: U-b & D-b Not different, U-b & U-a Not diff (p adj = 0.265), U-a & D-a Not different,

# pairwise.adonis(x=wisconsin(mv_fam.taxa[-(1:6),-30]),factors = mv_fam$Impact2[-c(1:6)],perm=9999)

library(pairwiseAdonis)
# with New sites excluded
m2<-adonis(mv_fam.taxa[-c(1:6,which(mv_fam$Impact2=="New")),-30]^0.25 ~ Impact2, data=mv_fam[-c(1:6,which(mv_fam$Impact2=="New")),], permutations=9999 ,method="bray")
print("New sites excluded")

## [1] "New sites excluded"

m2 # significant difference

##
## Call:
## adonis(formula = mv_fam.taxa[-c(1:6, which(mv_fam$Impact2 == "New")), -30]^0.25 ~ Impact2, data = mv_fam[-c(1:6, which(mv_fam$Impact2 == "New")), ], permutations = 9999, method = "bray")
##
## Permutation: free
## Number of permutations: 9999
##
## Terms added sequentially (first to last)
##
## Df SumsOfSqs MeanSqs F.Model R2 Pr(>F)
## Impact2 3 0.5809 0.193636 2.5609 0.16817 1e-04 ***
## Residuals 38 2.8733 0.075613 0.83183
## Total 41 3.4542 1.00000
## ---
## Signif. codes: 0 '***' 0.001 '**' 0.01 '*' 0.05 '.' 0.1 ' ' 1

out<-pairwise.adonis(x=mv_fam.taxa[-c(1:6,which(mv_fam$Impact2=="New")),-30]^0.25,factors = mv_fam$Impact2[-c(1:6,which(mv_fam$Impact2=="New"))],perm=9999)
# at alpha=0.01: D-b different from U-a
# at alpha=0.05: U-b different from D-a; D-b different from U-a, D-b diff from D-a; U-a & U-b Not different (p adj = 0.166)
out[,1]<-gsub(x = out$pairs,pattern = "stream",replacement = "",fixed=TRUE)
print("Fourth-root transformation")

## [1] "Fourth-root transformation"

format(out,digits=5)

## pairs Df SumsOfSqs F.Model R2 p.value p.adjusted
## 1 Up-before vs Down-before 1 0.19557 2.4766 0.198498 0.0209 0.1254
## 2 Up-before vs Up-after 1 0.17171 2.2200 0.181672 0.0291 0.1746
## 3 Up-before vs Down-after 1 0.21512 2.9154 0.108317 0.0039 0.0234
## 4 Down-before vs Up-after 1 0.24792 3.1485 0.183601 0.0005 0.0030
## 5 Down-before vs Down-after 1 0.21946 2.9264 0.094624 0.0031 0.0186
## 6 Up-after vs Down-after 1 0.12823 1.7233 0.057977 0.0768 0.4608
## sig
## 1
## 2
## 3 .
## 4 *
## 5 .
## 6

out<-pairwise.adonis(x=wisconsin(mv_fam.taxa[-c(1:6,which(mv_fam$Impact2=="New")),-30]),factors = mv_fam$Impact2[-c(1:6,which(mv_fam$Impact2=="New"))],perm=9999)
out[,1]<-gsub(x = out$pairs,pattern = "stream",replacement = "",fixed=TRUE)
print("Wisconsin standardization")

## [1] "Wisconsin standardization"

format(out,digits=5)

## pairs Df SumsOfSqs F.Model R2 p.value p.adjusted
## 1 Up-before vs Down-before 1 0.47375 2.2038 0.180585 0.0106 0.0636
## 2 Up-before vs Up-after 1 0.36264 1.4703 0.128181 0.1224 0.7344
## 3 Up-before vs Down-after 1 0.38722 1.6496 0.064312 0.0569 0.3414
## 4 Down-before vs Up-after 1 0.62495 2.5613 0.154656 0.0002 0.0012
## 5 Down-before vs Down-after 1 0.61345 2.6091 0.085240 0.0009 0.0054
## 6 Up-after vs Down-after 1 0.26659 1.0818 0.037199 0.3592 1.0000
## sig
## 1
## 2
## 3
## 4 *
## 5 *
## 6

Upstream sites before and after dam removal were not different (p adj = 0.265 and 0.166, with and without “New” sites, respectively, both using 4th root transformed data, and p adj > 0.75 using Wisconsin transformation).

With “New” sites included (4th root transformation), they are different from all the other groups (adj p<-.013), and Downstream-before sites are different from Upstream-after sites (adj p=0.003) and from Downstream-after sites (adj p=0.023). This result is consistent with our earlier findings of difference between Downstream sites before and after dam removal.

##
## Permutation test for homogeneity of multivariate dispersions
## Permutation: free
## Number of permutations: 999
##
## Response: Distances
## Df Sum Sq Mean Sq F N.Perm Pr(>F)
## Groups 3 0.033111 0.0110370 1.6455 999 0.195
## Residuals 47 0.315241 0.0067073
##
## Pairwise comparisons:
## (Observed p-value below diagonal, permuted p-value above diagonal)
## Upstream Downstream-before Downstream-after New
## Upstream 0.717000 0.586000 0.063
## Downstream-before 0.707869 0.969000 0.125
## Downstream-after 0.596156 0.966426 0.071
## New 0.046601 0.114723 0.068101

##
## Permutation test for homogeneity of multivariate dispersions
## Permutation: free
## Number of permutations: 999
##
## Response: Distances
## Df Sum Sq Mean Sq F N.Perm Pr(>F)
## Groups 1 0.019952 0.0199521 3.0174 999 0.109
## Residuals 13 0.085961 0.0066124

##
## Call:
## adonis(formula = mv_fam.taxa[nynu, -57]^0.25 ~ Impact, data = mv_fam[nynu, ], permutations = 999, method = "bray")
##
## Permutation: free
## Number of permutations: 999
##
## Terms added sequentially (first to last)
##
## Df SumsOfSqs MeanSqs F.Model R2 Pr(>F)
## Impact 1 0.16179 0.161792 2.8609 0.18038 0.007 **
## Residuals 13 0.73518 0.056552 0.81962
## Total 14 0.89697 1.00000
## ---
## Signif. codes: 0 '***' 0.001 '**' 0.01 '*' 0.05 '.' 0.1 ' ' 1

Looking at only the years 2013-2015, New sites are still under-dipsersed compared to the Downstream sites. Using PERMANOVA to compare New and Upstream sites shows that they are different (p=0.0066), but we can’t include the downstream sites here because they have different dispersion.

### Indicator Species

Goal is a panel plot with one panel per taxa, one line per site class (upstream, downsteam, new), x-axis is the years

The best indicators for each type of site are listed here - there are a total of 16, which seems like too many for a plot. Lets work with the top 1-2 for each type of site.

Chironomidae, Helicopsychidae, (significant from SIMPER and FFG analysis, but not indicator analysis), Baetidae, Philopotamidae, Brachycentridae, Bivalvia, and Gordiidae.(from indicator analysis). Also, divide all values by 0.558 to convert to density (individuals/m^2)

indic8<-c("Chironomidae","Helicopsychidae","Baetidae","Philopotamidae","Brachycentridae","unk_Bivalvia","Gordiidae")
which(names(mv_fam)%in%indic8)

## [1] 7 12 17 19 25 31 38

indic8<-mv_fam[,c(1,2,41,which(names(mv_fam)%in%indic8))]
# 57 observations for 8 taxa plus Year, Site, and Impact
# incid8$Impact<-mv_fam$Impact
indic8<-indic8[indic8$Year>2010,]
# remove pre 2010 data
indic8[,4:10]<-indic8[,4:10]/0.558 # scale to density/m^2
indic8<-as.data.frame(indic8) # away with ye, ye troublesome tibbles
## planning
apply(indic8[,4:10],MAR=2,max)

## Chironomidae Baetidae Brachycentridae Helicopsychidae Philopotamidae
## 469.534050 245.519713 68.100358 46.594982 5.376344
## unk_Bivalvia Gordiidae
## 5.376344 91.397849

# many taxa have single digit values, but Chironomidae goes to 262
# for 3x3 layout: Bivalvia,Philo,[Legend], Brachy, Gord, Helico, Baetidae, Chiron, Elmidae
table(indic8$Impact)

##
## Upstream Downstream-before Downstream-after New
## 12 8 22 9

# Will need to combine Down-before and Down-after
indic8$SiteClass<-indic8$Impact
levels(indic8$SiteClass)<-c("Upstream","Downstream","Downstream","NewChannel")

sem<-function(x){sd(x,na.rm=TRUE)/sqrt(sum(is.na(x)==FALSE))}

in8means<-aggregate(indic8[4:10],by=indic8[c(1,11)],mean,na.rm=TRUE)
in8sem<-aggregate(indic8[4:10],by=indic8[c(1,11)],sem)
in8semUp<-in8sem
in8semUp[,3:9]<-in8means[,3:9]+in8sem[,3:9]
in8semLo<-in8sem
in8semLo[,3:9]<-in8means[,3:9]-in8sem[,3:9]
in8sum<-rbind(in8means,in8semUp,in8semLo)
rm(in8semUp,in8semLo)
in8sum$Var<-rep(c("mean","seUp","seLo"),each=15)
in8sum<-in8sum[,c(1,2,10,3:9)] # same order of species

## quartz(width=6,height=6)
#save.image("~/Dropbox/Boardman River/Working Envt.RData")
# setup
# cols<-c("#D81B60","#FFC107","#004D40") # rm #2
cols<-c("grey60","#009E73","#E69F00") # "#0000000","#0072B2"
ylims<-c(475,475,70,70,6,6,95)
axNames<-names(in8sum);axNames[9]<-"Unknown Bivalvia"

#tiff(filename="IndTaxa.tif",1800,1800,units="px",compression = "lzw",res=300)
par(oma=c(4,2.5,1,2.5),cex=0.9)
layout(matrix(c(6,4,2, 8,3,1, 8,5,7),ncol=3))
for(p in 4:10){
 #### adjust margins
 if(p %in% c(4,6,8,10)){
 par(mar=c(0.4,0,1.5,0.4))
 } else{
 par(mar=c(0.4,0.4,1.5,0))
 }
 #### create plot ####
 fv=p #fv='focus variable'
 plot(indic8$Year,indic8[[fv]],type="n",xaxt="n",yaxt="n",xlim=c(2010.5 ,2016.5),xlab="",ylim=c(0,ylims[p-3])) # plot
 legend(x=c(2010.26,2016.74),y=ylims[p-3]*c(1.04,1.18),legend=axNames[p],cex=1,xpd=NA,bg="grey80") # label
 abline(v=2012.5,lty=2,col="grey20") # line of dam removal
 #### add points ####
 points(jitter(indic8$Year[indic8$SiteClass=="Upstream"], factor=0.7)-0.2, indic8[indic8$SiteClass=="Upstream",fv], bg=cols[1],pch=21,cex=0.7)
 points(jitter(indic8$Year[indic8$SiteClass=="Downstream"], factor=0.7)+0.0, indic8[indic8$SiteClass=="Downstream",fv], bg=cols[2],pch=21,cex=0.7)
 points(jitter(indic8$Year[indic8$SiteClass=="NewChannel"], factor=0.7)+0.2, indic8[indic8$SiteClass=="NewChannel",fv], bg=cols[3],pch=21,cex=0.7)

 #### add error bars ####
 arrows(x0=in8sum$Year[which(in8sum$SiteClass=="Upstream"&in8sum$Var=="mean")]-0.2, y0=in8sum[which(in8sum$SiteClass=="Upstream"&in8sum$Var=="seUp"),fv], y1=in8sum[which(in8sum$SiteClass=="Upstream"&in8sum$Var=="seLo"),fv], length=0,col=cols[1])
 arrows(x0=in8sum$Year[which(in8sum$SiteClass=="Downstream"&in8sum$Var=="mean")]+0.0, y0=in8sum[which(in8sum$SiteClass=="Downstream"&in8sum$Var=="seUp"),fv], y1=in8sum[which(in8sum$SiteClass=="Downstream"&in8sum$Var=="seLo"),fv], length=0,col=cols[2])
 arrows(x0=in8sum$Year[which(in8sum$SiteClass=="NewChannel"&in8sum$Var=="mean")]+0.2, y0=in8sum[which(in8sum$SiteClass=="NewChannel"&in8sum$Var=="seUp"),fv], y1=in8sum[which(in8sum$SiteClass=="NewChannel"&in8sum$Var=="seLo"),fv], length=0,col=cols[3])

 #### add lines ####
 lines(x=in8sum$Year[which(in8sum$SiteClass=="Upstream"&in8sum$Var=="mean")]-0.2, y=in8sum[which(in8sum$SiteClass=="Upstream"&in8sum$Var=="mean"),fv], col=cols[1])
 lines(x=in8sum$Year[which(in8sum$SiteClass=="Downstream"&in8sum$Var=="mean")]+0.0, y=in8sum[which(in8sum$SiteClass=="Downstream"&in8sum$Var=="mean"),fv], col=cols[2])
 lines(x=in8sum$Year[which(in8sum$SiteClass=="NewChannel"&in8sum$Var=="mean")]+0.2, y=in8sum[which(in8sum$SiteClass=="NewChannel"&in8sum$Var=="mean"),fv], col=cols[3])
 #### x axis labels ####
 if(p %in% c(4,5,10)){
 axis(side=1,cex.axis=0.7)
 mtext(side=1,line=2,"Year",cex=0.7)
 } # end xaxis if
 #### y axis labels ####
 if(p %in% c(5,7,9)){
 axis(side=2,cex.axis=0.9)}
 if(p %in% c(8,10)){
 axis(side=4,cex.axis=0.9)}
 # box(which="figure",col="red") # troubleshooting
} # end panel loop

# legend and labels ("plot 8")
par(mar=c(0.2,0.2,0,0.2))
plot.new()
legend("bottom",inset=0.2,pch=21,pt.bg=cols,col="black",legend = c("Upstream","Downstream","New Channel"),box.col="white",bg="white",cex=1.2)
legend("top",inset=0.1,legend = expression(paste("Density (Individuals ",m^{-1},")")),box.col="white",bg="white",xpd=NA,cex=1.2)


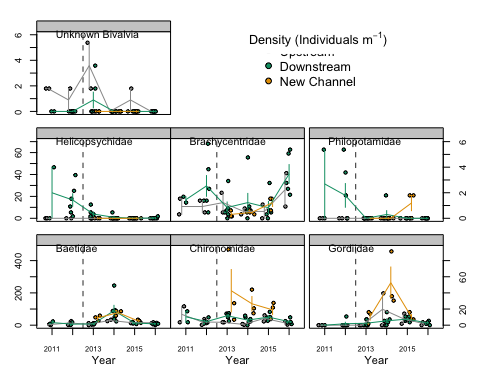

Supplement: S3 File — (DOCX) [file pone.0245030.s003.docx]
